# Intra-Host Diversity of SARS-Cov-2 Should Not Be Neglected: Case of the State of Victoria, Australia

**DOI:** 10.3390/v13010133

**Published:** 2021-01-19

**Authors:** Alix Armero, Nicolas Berthet, Jean-Christophe Avarre

**Affiliations:** 1 The Center for Microbes, Development and Health, Institut Pasteur of Shanghai—Chinese Academy of Sciences, Discovery and Molecular Characterization of Pathogens, Shanghai 200000, China; nicolas.berthet@pasteur.fr; 2 ISEM, University of Montpellier, CNRS, EPHE, IRD, 34095 Montpellier, France; jean-christophe.avarre@ird.fr

**Keywords:** SARS-CoV-2, quasispecies, nonsynonymous iSNVs, transmission, phylogenetic, genetic bottleneck

## Abstract

Since the identification of severe acute respiratory syndrome coronavirus 2 (SARS-CoV-2) as the etiological agent of the current COVID-19 pandemic, a rapid and massive effort has been made to obtain the genomic sequences of this virus to monitor (in near real time) the phylodynamic and diversity of this new pathogen. However, less attention has been given to the assessment of intra-host diversity. RNA viruses such as SARS-CoV-2 inhabit the host as a population of variants called quasispecies. We studied the quasispecies diversity in four of the main SARS-CoV-2 genes (ORF1a, ORF1b, S and N genes), using a dataset consisting of 210 next-generation sequencing (NGS) samples collected between January and early April of 2020 in the State of Victoria, Australia. We found evidence of quasispecies diversity in 68% of the samples, 76% of which was nonsynonymous variants with a higher density in the spike (S) glycoprotein and ORF1a genes. About one-third of the nonsynonymous intra-host variants were shared among the samples, suggesting host-to-host transmission. Quasispecies diversity changed over time. Phylogenetic analysis showed that some of the intra-host single-nucleotide variants (iSNVs) were restricted to specific lineages, highlighting their potential importance in the epidemiology of this virus. A greater effort must be made to determine the magnitude of the genetic bottleneck during transmission and the epidemiological and/or evolutionary factors that may play a role in the changes in the diversity of quasispecies over time.

## 1. Introduction

In December 2019, China notified the World Health Organization (WHO) that several people with severe pneumonia had been admitted to an intensive care unit at Jin-Yin-Tan Hospital in Wuhan City in Chinese Hubei Province [1,2,3]. It was soon established that these patients were infected with a virus never observed in humans before. This novel coronavirus, severe acute respiratory syndrome coronavirus 2 (SARS-CoV-2), which belongs to family Coronaviridae, genus Betacoronavirus and subgenus Sarbecovirus, has a positive single-stranded RNA linear genome of 29.9 kb [1,2,3,4,5,6]. Together with the SARS-CoV and MERS-CoV, SARS-CoV-2 is one of the CoVs that can cause severe disease in the human population [7]. Betacoronaviruses, such as SARS-CoV or MERS-CoV, have a propensity for host jumping from various mammal species to humans. Similar to these other coronaviruses, SARS-CoV-2 certainly has a zoonotic origin, sharing 96.2% identity with a CoV bat strain, RaTG13, which has been found in horseshoe bats [1]. 

Given the current situation, it is essential to monitor the diversity of this new human pathogen and its potential implications for pathogenicity and infectivity. The diversity of SARS-CoV-2 is the result of the conjunction of patterns of variability at the population and the intra-host levels, which are products of selective, stochastic and spatio-temporal processes [8]. 

The phylogenetic analysis of the consensus genomic sequences of SARS-CoV-2 obtained from around the globe reveals a structure determined by geographical and temporal patterns of transmission. The largest clade in the SARS-CoV-2 phylogeny is defined by the presence of the 614G mutation in the spike (S) glycoprotein [9]. The SARS-CoV-2 S protein binds to the angiotensin-converting enzyme 2 (ACE2) on the surface of the human cell membrane mediating the fusion and entry of the virus. The study of the evolution of this protein in the outbreaks of other coronaviruses suggests that it plays a major role in the interspecies jump and in the adaptation to the ACE2 receptor, determining the infectivity of the virus [10,11]. In various countries, the viral sequences bearing the D614G mutation have become predominant after introduction, suggesting an adaptive advantage related to infectivity [12,13,14]. However, these conclusions have been contested in other studies [15,16].

Another gene responsible for the current phylogenetic structuring of SARS-CoV-2 is ORF1. This gene is composed of two open reading frames (ORFs) (a and b) coding for 16 nonstructural proteins (nsps) that compose the viral replication–transcription complex, including the RNA-dependent RNA polymerase (nsp12), [17]. ORF1 shows the highest number of missense mutations in SARS-CoV-2 [9], mainly in the nsp3 gene, a pattern that has also been observed in MERS-CoV [17]. One of the main mutations identified in the SARS-CoV-2 ORF1b gene is P314L, which occurs simultaneously with the D614G mutation of the S gene [9]. It is expected that the ORF1 gene acquired the adaptive mutations necessary to adjust the viral replication machinery to the new host, as shown in the adaptation of avian influenza virus to mammalian hosts [18,19]. Therefore, the P314L mutation may accelerate viral replication [9]. However, to our knowledge, no studies have addressed this hypothesis.

The nucleocapsid (N) protein is a structural protein involved in packaging the viral RNA [20]. The nucleocapsid of SARS-CoV-2, together with the S protein, modulates the antibody response [21]. Two important mutations in this gene, R203K and G204R, are found in sequences carrying the D614G mutation in the S gene and the P314L mutation in the ORF1b gene. Therefore, these mutations may be related to the interaction of the N protein with the membrane protein of SARS-CoV-2 [22]. 

The intra-host diversity of RNA viruses is associated with the quasispecies concept. A quasispecies is a cloud of diverse variants that are genetically linked through mutation, that interact cooperatively on a functional level, and that collectively contribute to the characteristics of the population [23]. Deep sequencing has revealed evidence of quasispecies in SARS-CoV [24,25,26,27] and MERS-CoV [28,29,30,31]. This intra-host diversity contributes to the adaptation of these viruses to the human host. Analysis of the MERS-CoV sequence shows an out-of-frame deletion, leading to the loss of a large part of the S2 subunit of S protein and resulting in the production of a shortened protein bearing only 801 amino acids. Although this deletion is expected to lead to the production of defective viruses, alternatively, this mutation may block spike-specific MERS-CoV neutralizing antibodies [32]. During the outbreak of MERS-CoV in the Republic of Korea in 2015, the virus presenting the D510G and I529T mutations at different intra-host frequencies in the receptor-binding domain (RBD) of the S protein showed increased resistance against neutralizing monoclonal antibodies and a reduced sensitivity to antibody-mediated neutralization [29].

A major issue in the current pandemic is to determine the diversity of the SARS-CoV-2 quasispecies and its potential contributions to population diversity and virus adaptation. We therefore studied the dynamics of the diversity of intra-host variants over a six-week period using public next-generation sequencing (NGS) SARS-CoV-2 sequences from the State of Victoria (Australia). The diversity of SARS-CoV-2 in Victoria is a snapshot of global diversity, because (i) most infected patients acquired the virus abroad and imported it into Australia and (ii) the epidemiological analysis shows that onward transmission of the contagion was limited [33]. 

We analyzed the intra-host diversity of the S, N and ORF1 genes of 210 samples from the State of Victoria collected between February and April 2020. First, we described the frequency and presence of synonymous and nonsynonymous intra-host single-nucleotide variants (iSNVs) in the SARS-CoV-2 genes. Then, we studied the changes in the diversity of shared iSNVs over time. Finally, we analyzed the distribution of the diversity of iSNVs in the different clades of the phylogenetic tree of consensus sequences. Our results show evidence of iSNVs transmission, and modification over time in this diversity.

## 2. Materials and Methods

### 2.1. Samples

In total, 217 samples of PRJNA613958 BioProject and related metadata were recovered from the NCBI website using the SRA-Toolkit (http://ncbi.github.io/sra-tools/). This BioProject involves more than 1000 Australian NGS samples from the State of Victoria. Our dataset represents a subsample of this BioProject obtained between 31 January 2020 and 8 April 2020. Our selection criteria involved sequences obtained using NextSeq 550 technology only.

### 2.2. Identification of iSNVs

iSNVs were identified in the ORF1a, ORF1b, S and N genes of the SARS-CoV-2 genome in each of the samples. We considered iSNVs to be those with a median alternative allele frequency (AAF) between 5% and 50%. The bioinformatics pipeline involved the following steps: low-quality read trimming with Trimmomatic [34]; alignment of the reads using Bowtie2 [35] with the SARS-CoV-2 reference sequence [3]; conversion of the same file alignments to bam files using samtool [36]; sorting the bam files and removing duplicate sequences with MarkDuplicate (http://broadinstitute.github.io/picard/). ViVarSeq [37] scripts derived a consensus sequence from the alignments obtained in the last step. The trimmed reads were realigned to the consensus sequence using Bowtie2. VirVarSeq identified the variants and their frequencies. This pipeline was semi-automated with Snakemake [38].

In each of the genes, the iSNVs were identified only in samples having a minimum coverage of 30 reads for 90% of the positions analyzed. Genomic positions with less than 30 reads and/or a Phred score lower than 20 were discarded for variant identification. We only considered variants supported by at least 5 reads. In parallel, we identified iSNVs in the SARS-CoV-2 genome with the V-Phaser2 [39]. Only iSNVs satisfying the aforementioned quality criteria and also identified by V-Phaser2 were included in the analysis.

Overall, 210 of these samples matched our criteria for at least one of the SARS-CoV-2 genes under analysis, 5 samples were eliminated for not presenting sufficient coverage and 2 for presenting more than 100 variants (outliers). Figure S1 presents the coverage and depth of the four SARS-CoV-2 genes in the 210 samples. The median of reads for each of the positions varied between 92 and 3100 for the S gene, 76 and 4160 for ORF1a, 392 and 4324 for ORF1b and from 318 to 2987 for the N gene. The median Phred score in the positions with iSNVs was 34 (IQR (33–35)) and the median number of reads representing a specific iSNV was 40 (IQR (23–120)). 

### 2.3. Temporal Dynamics of iSNV Diversity

The patterns of temporal variation in the diversity of synonymous and nonsynonymous iSNVs were represented using the ggplot2 [40] and EvoFreq [41] packages.

### 2.4. Identification of the iSNV Haplotypes

To determine if nonsynonymous iSNVs cosegregate in the same sequences (haplotypes), all reads spanning the specific region of the SARS-CoV-2 genome containing these variants were identified using the pysam (https://pysam.readthedocs.io/en/latest/faq.html) module of Python from the alignment of reads to the reference sequence of this virus. At each position of the region of interest, the reads and the respective nucleotides were identified. With this information, we determined the proportion of the reads that carried the combination of variants of interest.

### 2.5. Validation of iSNVs Haplotypes with Other Sequence Datasets

The existence of the viral haplotypes in the S gene was verified in two additional sample subsets available at NCBI: 232 samples from PRJNA625551 BioProject and 120 samples from PRJNA610428 BioProject.

### 2.6. Phylogenetic Analysis

For this analysis, 863 sequences of SARS-CoV-2 from Victoria were recovered on 29 June 2020 from GISAID [42] using the criteria of complete sequence and exclusion of low coverage. To improve the temporal signal of the phylogenetic analysis, 14 sequences from the Wuhan region collected during the month of January 2020 and the reference sequence from SARS-CoV-2 were also recovered from the GISAID website. The sequences of the ORF1, S and N genes were concatenated and aligned using MAFFT [43]. These alignments were visually inspected with Unipro UGENE [44]. The phylogenetic and temporal signal of this alignment was analyzed according to the guidelines suggested in Mavian et al. [45]. The phylogenetic signal was evaluated using iqtree [46] with the likelihood mapping analysis. To explore the presence of a temporal signal, a phylogenetic reconstruction was applied using iqtree [47,48] software with the -m option grouped to TEST, allowing the identification of the best model for partitions representing the four genes and a bootstrap analysis of 1000 replicates. The outliers were identified in this phylogenetic tree with the TempEst [49] software, using the regression analysis of the phylogenetic distance of the tips to the root and the collection time. This pruned tree was scaled with treedater [50] software using a strict molecular clock. The phylogenetic clusters were annotated using Pangolin software (https://github.com/cov-lineages/pangolin).

### 2.7. SNVs Identification

Single nucleotide variants (SNVs) in GISAID and NGS consensus sequences were identified from the multiple alignment with the QSutils [51] package in R. Mutations present in at least 1% of the samples were included in the study.

## 3. Results

### 3.1. Synonymous and Nonsynonymous iSNVs

A total of 493 iSNVs (representing 1153 occurrences) were identified in 68% (142/210) of the patient samples (Table S1). The median number of iSNVs per sample was one with a range between 0 and 45. The S gene had the highest mean density of iSNVs (4.41 × 10^−4^), followed by the ORF1a gene (1.84 × 10^−4^); the ORF1b and N genes showed similar mean densities (1.68 × 10^−4^ and 1.67 × 10^−4^, respectively). The main substitutions were G > T (22%, 107), followed by C > T (18%, 86), T > C (12%, 60), A > G (11%, 53) and G > A (10%, 49). The G > T substitution was dominant in the ORF1a (64), S (13) and N (8) genes, but C > T was dominant in the ORF1b gene (27), (Table S2). The median alternative allele frequency (AAF) was 7.44%, ranging from 5% to 48.93%.

Among the 493 iSNVs, 24% (119, representing 135 occurrences) were synonymous and 76% (374, 1018 occurrences) were nonsynonymous. The nonsynonymous iSNVs resulted in 324 (723 occurrences) amino-acid substitutions, 16 of these were stop codons. The median number of nonsynonymous iSNVs per sample was one (0–44), and the median number of synonymous iSNVs was zero (0–10). The highest mean of nonsynonymous variant density was observed in the S gene (4.26 × 10^−4^), followed by ORF1a (1.57 × 10^−4^), ORF1b (1.42 × 10^−4^) and the N gene (1.38 × 10^−4^). The distribution of nonsynonymous substitution density was significantly wider than that of the synonymous substitution density in all genes (Wilcoxon signed-rank test, *p* < 0.05, Figure S2).

### 3.2. Changes in iSNVs Diversity over Time

#### 3.2.1. Nonsynonymous Substitutions

Only 28% (103/374) of the nonsynonymous variants were observed in at least two samples. However, these variants represented 75% (763/1018) of the observed substitution occurrences.

**Figure 1:**
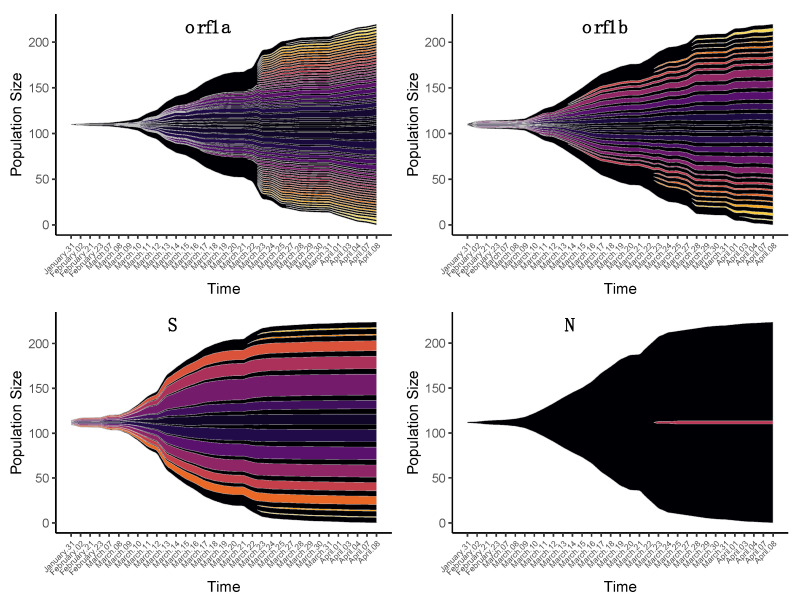
Temporal patterns of the diversity of nonsynonymous intra-host single-nucleotide variants (iSNVs) in four SARS-CoV-2 genes. Each fish plot represents the proportion of a specific nonsynonymous iSNV in the time interval covered by the samples. The dates indicate the days of sample collection.

Two different events of diversity in the ORF1 and S genes were identified when the proportion of nonsynonymous iSNVs was studied over time (Figure 1). One group of variants, observed since early February, showed a peak in frequency between 15 and 20 March. Another group corresponded to variants that arose mainly after 23 March. For simplicity, the first group of variants was called “Early” and the substitutions that emerged in late March were called “Late”. These mutations and the proportion of samples in which they were observed are given in Table S3. 

**Figure 2:**
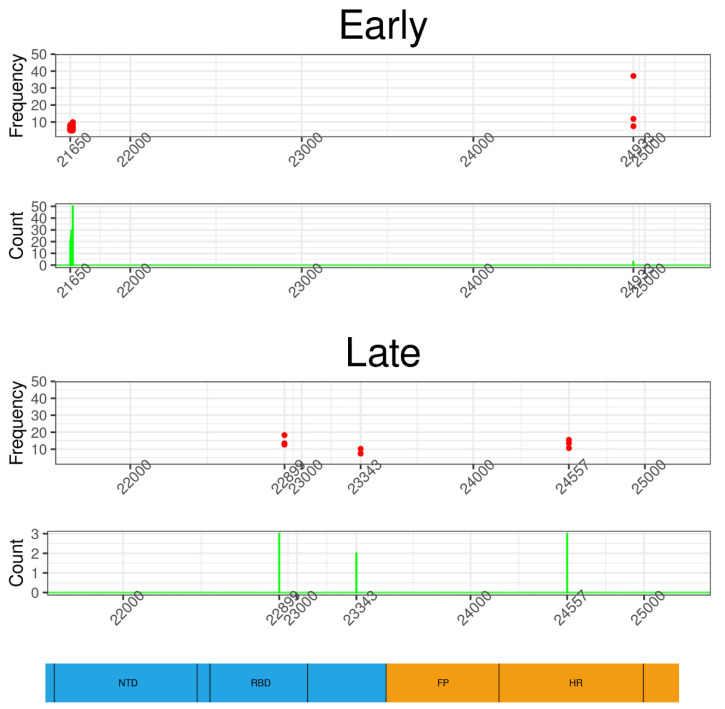
Genomic distribution of nonsynonymous iSNVs of two temporal groups in the S gene. Each dot represents the occurrence and frequency of a specific variant from Early and Late groups. The majority of iSNVs of the Early group are concentrated in a small genomic region starting at position 21,650; they have low intra-host frequencies but are shared by a significant number of samples. The variants of the “Late” group are observed at three positions, one of them localized in the receptor-binding domain (RBD); these mutations have higher frequencies but are shared by a small number of samples. The bottom of the figure shows a schematic representation of the S gene and its main domains: N-terminal domain (NTD), receptor-binding domain (RBD), fusion peptide (FP), heptad repeat (HR).

The genomic distribution of the nonsynonymous variants of the two different temporal groups showed different patterns in the S gene (Figure 2). The majority of Early nonsynonymous iSNVs of the S gene were concentrated in a small region. Of the 11 nonsynonymous iSNVs identified in the Early group, 10 were located between genomic positions 21,650 and 21,665. These variants concentrated 99% (276/279) of the occurrences identified in this group. The translation of these variations led to N30G, S31Stop, F32Stop, R34P and G35R amino-acid substitutions. To explore whether these variations were carried by the same viral genomic sequence, i.e., haplotype, we recovered the reads that completely spanned this region and determined the frequencies at which these variations were found together. To distinguish this process from AAF, we refer to it as haplotype frequency. The N30G/S31Stop/F32Stop/R34P/G35R haplotype (haplotype frequency >5%) was found in 24% (47/195) of samples in which the S gene sequences were analyzed, with a median haplotype frequency of 6.2%. We found a strong correlation between haplotype frequency and the AAF of individual variants, indicating that these mutations co-segregate in the same haplotype in most cases (Table S4). The Late nonsynonymous iSNVs were found at three different positions along the S gene presenting AAFs of less than 20% (Figure 2). The G22899T variant, identified in three samples, leads to the G446V amino-acid substitution in the RBD domain of the S protein (Figure 2). In this group, the G24557T variant leading to the amino acid G999C was also located in the heptad repeat (HR) region of the S protein. 

The tendency to form haplotypes in the Early group was also observed in the ORF1 gene (Figures S3 and S4). Eight different variants between positions 2822 and 2833 formed a haplotype involving the L853Stop, N854A and K856H substitutions in ORF1a. This haplotype was observed in 13% (28/208) of the samples, with a relatively high median haplotype frequency (19.6%); eight samples of this group had haplotype frequencies higher than 15%, reaching up to 27.6% (Figure S3).

In ORF1b, two viral haplotypes were also observed. The first one involved the amino-acid substitutions E1120V, Y1121L and T1122S, resulting from five nucleotide variants between positions 16,826 and 16,831. This haplotype was observed in less than 3% (6/207) of the samples with a median intra-host frequency of 7.99%. The second haplotype corresponded to positions 19,930–19,947 and included nine nucleotide variants that translated to M2155V, T2156S, D2157H, I2158R, A2159T and K2160N amino-acid substitutions. This haplotype was observed in 8.2% (17/207) of the samples, with a median intra-host frequency of 7.56%. 

Similar to what was observed in the S gene, the Late group iSNVs of ORF1 gene presented a wider distribution than the Early group variants (Figures S3 and S4). ORF1a presented 34 variants in the Late group, representing 32 different amino-acid substitutions. These substitutions were observed in between 1% and 2% of the samples. Some of these variants had AAFs higher than 20% in more than one sample, as was the case of G9141T, C13381A and T13380C. In ORF1b, 11 nucleotide variants involving nine amino-acid substitutions were identified in less than 3% of samples.

The gene encoding the SARS-CoV-2 N protein had no nonsynonymous iSNVs shared in the Early group. In the Late group, only one nucleotide substitution (G28559T) was observed in three patient samples, with an AAF lower than 9%, leading to the 96C amino-acid substitution.

#### 3.2.2. Synonymous Substitutions

Synonymous substitutions that were shared by at least two patients only occurred in ORF1a and ORF1b, in the Early and Late groups. For these genes, 5.88% (7/119) of synonymous iSNVs, representing 17.04% (23/135) of occurrences, were shared in at least two samples. ORF1a had three synonymous variants (T9223C, T8782C, T2839A) shared in the Early group and one variant (G13240T) shared in the Late group. Between these variants, T8782C had a median AAF of 18.21%, and the T2839A variant was shared by eight samples. ORF1b had two variants (T14805C and C17550T) shared in the Early group with a median AAF of 20.15%, and an iSNV (A16824T) in the Late group with a median AAF of 5.79%. None of the synonymous variants identified in the S and N genes were present in more than one patient.

#### 3.2.3. Origin of iSNVs in the Late Group

To investigate the possible causes of the new diversity in iSNVs observed in the Late group, we evaluated whether the samples with iSNVs in this group were associated with patients who imported the virus into Australia or with patients infected locally. In the dataset, 150 samples came from imported cases, 47 were contaminated locally, and no information was available for the remaining 13 samples. In total, 48.7% (73/150) and 40.4% (19/47) of the patients from imported and local cases, respectively, carried iSNVs of the Early and/or Late group. Of the patients who acquired the virus outside Australia, all (73/73) harbored Early-group iSNVs and 6.84% (5/73) harbored at least one Late-group variant. Among the patients infected locally, 89.5% (17/19) carried Early-group variants and 42.1% (8/19) carried Late-group variants. There was no significant association between the number of samples that carried Early-group variants and whether the patient acquired the virus abroad or locally (chi-squared test value, df, *p*-value = 0.055). However, the number of samples with Late-group iSNVs was significantly associated with the local or abroad acquisition of the virus (chi-square test, *p*-value < 0.05). The residuals of chi-square test showed a strong association between transmission at the local level and the fact that the sample presents iSNVs of the Late group (3.24).

### 3.3. Phylogenetic Analysis of Consensus Sequences and iSNVs

To better understand the role and dynamics of iSNVs in the evolution of SARS-CoV-2 in Victoria, we performed a phylogenetic analysis on viral consensus sequences. To obtain a sufficiently strong temporal and phylogenetic signal, we aligned the consensus sequences of the 210 NGS samples investigated here with 863 other sequences obtained in Victoria between January and June and with 14 sequences from the Wuhan region collected in December and January, all available on the GISAID website. A maximum-likelihood tree was constructed and outliers affecting the temporal signal were identified and eliminated. This final tree was composed of 717 sequences, of which 184 corresponded to the consensus sequences of the present NGS data. The regression coefficient of the evolutionary distance with respect to the collection date was 0.16, indicating the presence of a temporal signal. The tree was scaled relative to collection time. 

**Figure 3:**
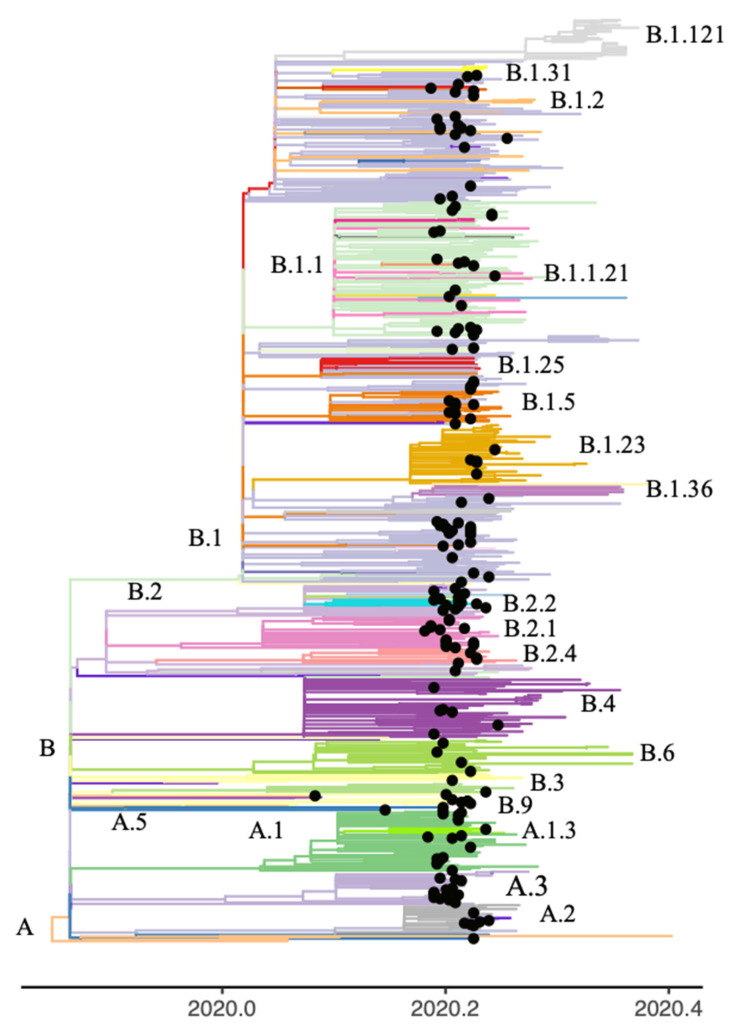
Phylogenetic tree of the severe acute respiratory syndrome coronavirus 2 (SARS-CoV-2) genomic sequences collected in the State of Victoria, Australia. The clusters are colored according to Pangolin annotation. The black tips represent the consensus sequences derived from the next-generation sequencing (NGS) data.

The main clusters were identified from single-nucleotide variants (SNVs) and the Pangolin annotation on the GISAID and consensus sequences. There were two major clusters defined by SNVs in the 614 and 314 positions of the S and ORF1b genes, respectively. The first cluster was defined by the concomitant presence of the D614 and P314 mutations (30–38% bootstrap support). All Wuhan sequences were found within this cluster. The second major cluster contained sequences with the G614 and L314 mutations (70% bootstrap support). Forty percent (74/184) of the NGS consensus sequences present in the phylogenetic tree had at least one iSNV from the Early and/or Late group (Figure 3). Among the 43 Early-group nonsynonymous iSNVs identified in the NGS samples present in the phylogenetic tree, 81% (35/43) were shared by the two major phylogenetic clusters, 14% (6/43) were exclusive to the D614/P314 cluster and 5% (2/43) to the G614/L314 cluster. None of the Early-group synonymous variants were shared between the two major clusters. In the Late group, 57% (27/47) of the nonsynonymous variants were shared by the two major clusters, whereas 34% (16/47) were exclusive to the G614/L314 cluster and 9% (4/47) to the D614/P314 cluster. A Late-group synonymous variant was shared by sequences from the two major clusters.

The variants that were exclusive to one of the largest clades, were preferentially observed in the same Pangolin lineage or in a sister lineage. For example, among the Early group variants, the T9223C, T9476A and T9477C mutations of ORF1a were observed exclusively among samples of Pangolin lineage B.2.2.2. While variant T14805C of ORF1b and T11083G of ORF1a were identified in lineage B.2 and derivative sublineage B.2.4. Interestingly, the G3464T ORF1a and G24933T S variants were observed in the B.1 lineage at intra-host frequencies below 20% and at frequencies close to 40% in one sample from B.1.13 lineage (Figure S5).

## 4. Discussion

### 4.1. Diversity of SARS-CoV-2 iSNVs

Our analysis showed that a significant percentage of the SARS-CoV-2 sequences of the S, N and ORF1 genes bore evidence of quasispecies diversity. This result corroborates previous studies that showed intra-host variability during epidemic outbreaks of SARS-CoV [24,25,26,27], MERS-CoV [28,29,30,31] and more recently SARS-CoV-2 [52,53,54,55]. The median number of iSNVs per patient estimated in our study was low in contrast to previous studies in which a high number of minority variants were estimated per sample [52,53,54,55]. However, our results were consistent with an extensive analysis of the available NGS data for SARS-CoV-2 from the NBCI [53]. We believe that these differences are due to sequencing strategies, bioinformatics pipelines and filtering criteria that can lead to this type of discrepancy [56]. Here, we opted for a conservative approach where only iSNVs identified using two different strategies were selected.

We found that the most frequent intra-host substitutions in Victoria are G > T, C > T, T > C and A > G. Previous studies have shown the important role that C > T and T > C mutations play in the dynamics of SNVs in consensus sequences of SARS-CoV-2. The C > T substitutions can be the product of a host defense mechanism mediated by enzymes of the APOBEC3 family [57,58]. A comparison of the intra- and inter-host substitution patterns in samples from two different cities in the United States found a high prevalence of C > T substitutions (except for intra-host diversity in one city) [54]. The G > T transversion is the third most common type of substitution in the consensus sequences of SARS-CoV-2 worldwide, but in Asia and Oceania it is the second most common type [59]. Likewise, our results indicate that the most frequent nucleotide substitutions were G > T and C > T, suggesting local differences in substitution patterns. The high frequency of G > T transversion in SARS-CoV-2 sequences is striking, because transitions are more likely than transversions [60]. This transversion is probably initiated by 8-oxoguanine derived from a reactive oxygen species [61], implying the active role of oxidative stress in the emergence of this variation, a hypothesis that needs further investigation.

We focused our analysis on the genes that played a major role in the adaptation of SARS-CoV [24] and MERS-CoV [28,29,30] to the human host, and those in SARS-CoV-2 that modulate the antibody response [21]. The majority of iSNVs in these genes were nonsynonymous. This predominance of nonsynonymous substitutions has already been documented for SARS-CoV-2, both at the consensus sequence level [57,58], and at the intra-host level [53,54]. As mentioned above, the C > T and G > T substitutions were predominant in the dataset. Because at least C > T may be the product of the action of the host’s enzymatic defense system, most nonsynonymous mutations in SARS-CoV-2 likely do not involve a selective advantage.

Although a significant proportion of SARS-CoV-2 quasispecies diversity may not represent adaptive variation, the virus is probably under selective pressure as a result of the interspecies jump to a new host. This adaptive process should be particularly evident in the proteins involved in the pathogenicity of the virus and in non-structural proteins that interact with the host’s immune system. Our data showed that the S gene has the highest density of nonsynonymous variants of the four genes analyzed. The amino-acid substitution G446V was identified in the RBD domain of the S protein involved in binding to the human ACE2 receptor. In addition, the G999C mutation was observed in the HR regions involved in membrane fusion during virus entry into the host cell. Zhang et al. suggest that the RBD domain and the HR regions played a determining role in the adaptation of SARS-CoV to the human host [10]. These authors identified two groups of amino acid sites under positive selection in consensus sequences: one related to the interspecies jump, mainly present in the RBD domain, and the other, involved in the adaptation to the new host and abundant in the HR region. Because our samples derive from the early stage of the adaptation of SARS-CoV-2 to the human host, it is difficult to affirm the adaptive value of the iSNVs identified in the important functional regions of the S gene. However, the emergence of this variability in regions critical for the pathogenicity of the virus requires spatial and temporal tracking.

The ORF1a gene showed a significant number of nonsynonymous iSNVs in the Late group, and some of these substitutions had AAFs greater than 20%. There is evidence for broad positive selection acting on the MERS-CoV ORF1a [17]. This selective pressure on a gene encoding non-structural proteins may be related to the interaction of these proteins with the human immune system. Alternatively, the replication machinery encoded by the ORF1a gene may be an essential element in the adaptation of the virus to its new host, as established for the adaptation of avian influenza A viruses to mammalian hosts [18]. 

The N gene had few nonsynonymous iSNVs. We identified the A29039T variant that led to the substitution of lysine by a stop codon at position 256. A previous analysis that characterized evolution of the viral lineages and transmission in SARS-CoV-2, considering both the consensus information and the iSNVs, also found the A29039T variant in a significant proportion of the samples analyzed. This concordance of results raises a red flag in regard to the efficacy of a SARS-CoV-2 vaccine directed against the N protein, because the stop codon produced by A29039T affects the linker region suppressing the immunogenic domain of this protein [62].

### 4.2. Viral Haplotypes and Quasispecies

Recent studies have demonstrated the presence of different haplotypes when comparing the diversity between the respiratory system and the intestinal tract [63,64]. Here, we identified four potential viral haplotypes in the investigated SARS-CoV-2 genes. Since we could not experimentally confirm the presence of these haplotypes, e.g., by digital PCR [65], we verified the presence of the most unexpected one, the N30G/S31Stop/F32Stop/R34P/G35R haplotype of the S gene, in a subset of samples from a North American cohort. This haplotype was found in 10 of the 232 analyzed samples that were collected in the same time period as the Australian ones, with a frequency ranging from 3% to 32% (Table S5). Both American and Australian sequences were obtained with the ARTIC PCR-tiling strategy, which involves a high number of overlapping amplicons of ~400bp [66]. We observed that this haplotype fell within the target region of one of the 218 ARTIC primers. It has been suggested that non-removal of primers from sequencing reads could lead to an underestimation of the frequency of iSNVs [67]. Trimming of the ARTIC primers from the reads did not affect the identification and frequency of the N30G/S31Stop/F32Stop/R34P/G35R haplotype in the S gene (Table S6). In order to evaluate a potential bias related to the ARTIC procedure, we analyzed 120 additional samples from another dataset obtained by metagenomics. In this dataset, we were unable to recover any of the mutations that are part of the proposed haplotype in the S gene. However, sequencing depth was much lower in these data, and we noted a significant number of iSNVs per sample in this cohort, suggesting a poor quality of sequencing data. Such a low sequencing depth makes it unsuitable for the identification of minority variants, possibly explaining the non-identification of the haplotype (data not shown). We also verified that the haplotype did not fall into a region known to be prone to Illumina sequencing artifacts [68], as the ARTIC procedure was applied on all samples of the Australian cohort. Therefore, if it cannot be ruled out that this potential haplotype results from sequencing artifacts linked to the ARTIC amplicon strategy, its asymmetric distribution between the Early and Late groups of variants remains difficult to explain, as the ARTIC strategy was applied on all samples of the Australian cohort.

Andrés et al. (2020) identified several deletions upstream of the S1/S2 cleavage site of S protein, in a study that included patients with mild and severe COVID-19 symptoms. These deletions were present at low frequencies and led to in-frame stop codons. The presence of stop codons close to the cleavage site of S1/S2 led to the loss of S2 translation. The authors proposed that the S1 subunit produced by this defective haplotype is released as a free protein in the extracellular space. This free S1 protein could bind to the human ACE2 cell receptor, thereby competing with complete viral particles and reducing the severity of infection. In this scenario, transmission of the haplotypes bearing deletions represents a selective advantage since attenuation of the infection increases viral transmission [69]. However, this study does not propose a molecular scenario to understand how these haplotypes with only S1 subunit are transmitted. Our analysis identified several other haplotypes with high frequencies, such as 853Stop/854A/856H in the ORF1a gene (~27%), supporting previous findings and raising the question of the role of these potential defective viral haplotypes within the quasispecies. Further experimental research is necessary to evaluate these hypotheses.

### 4.3. Changes in SARS-CoV-2 iSNVs Diversity over Time

We observed that the diversity of SARS-CoV-2 iSNVs changed over time, between patients. This change implied the emergence of a more heterogeneous pattern of diversity (Late group) that occasionally affected important antigenic regions of the virus proteins. The advent of this so-called Late group was concomitant to the epidemic peak in the State of Victoria and the related public health actions, such as the closing of the Australian border and the declaration of a state of emergency [33]. The Late-group population was enriched in patients who had acquired the virus through local transmissions. This relationship does not imply causality, and caution must be taken given the limited epidemiological and clinical information included in our analysis. Other epidemiological variables and information from transmission clusters may help clarify the emergence of diversity observed in the Late group. It was shown that patients with severe COVID-19 symptoms present a more important intra-host diversity than patients with mild symptoms [70]. Furthermore, Kuipers et al. established that age is significantly associated with intra-host viral genetic diversity [71]. The identification of these factors inducing new diversity is worth exploring in an in-depth analysis of existing genomic data integrated with extensive epidemiological and clinical information.

### 4.4. Transmission and Bottleneck of SARS-CoV-2 iSNVs

Almost one-third of SARS-CoV-2 quasispecies diversity in Victoria was shared between patients, suggesting host-to-host transmission. If potential artifacts are excluded, each iSNV would be shared by a median of three patients. Different studies suggest a relatively important genetic bottleneck in SARS-CoV-2 [55,64,72,73]. By analyzing the intra-host diversity of the S gene in two transmission clusters, Sun et al. evidenced a significant bottleneck that would lead to only 6% of the variants being stably transmitted [72]. Such a narrow bottleneck has also been demonstrated in the analysis of household transmission, which also suggested that this transmission is governed by stochastic processes [73]. Our results suggest that although transmission may be limited, this process does not seem to be random. Less than one-third of the nonsynonymous variants were shared between samples; however, they represented 70% of the total number of occurrences. This nonrandom transmission of quasispecies diversity has been observed in other RNA viruses [74,75], and some of these variants could be expected to have played a role in the response of the virus to the immune system.

Other factors may—at least partially—explain the shared diversity patterns of SARS-CoV-2 in Victoria. As suggested above, the action of APOBEC3 enzymes may lead to characteristic neutral or deleterious nucleotide substitution profiles in SARS-CoV-2. These enzymes act in specific sequence contexts, which causes the recurrence of substitutions, as suggested in SARS-CoV-2 genomic sequences [16]. It is possible that a part of the iSNVs diversity shared among patients is the product of this recurrence mediated by the host defense system. This hypothesis could explain the presence of the same iSNVs in different phylogenetic clades. In contrast, some of the SNVs identified in the current study were shared exclusively by patients with the same or a close Pangolin lineage. Then, it is necessary to determine to what extent the diversity of iSNVs is due to transmission between patients or to de novo intra-host mechanisms in SARS-CoV-2. Investigating whether iSNVs are transmitted, generated de novo or both, requires a large-scale longitudinal analysis of the evolution of intra and inter-host variability. It is likely that both transmission and de novo generation contribute to the diversity of SARS-CoV-2 quasispecies.

Here, we found evidence of intra-host quasispecies diversity in the NGS sequences of SARS-CoV-2 sampled in Victoria. This diversity was dynamic in time and possibly part of this variation was transmitted during the first epidemic episode in this Australian state.

## Data Availability

The pipeline for iSNVs identification is available in https://github.com/alexarmerov/SARS-CoV-2.

## Supplementary Materials

The following are available online at https://www.mdpi.com/1999-4915/13/1/133/s1, Figure S1: Coverage and depth of 210 SARS-CoV-2 samples, Figure S2: Comparison of the density distributions of synonymous and nonsynonymous iSNVs in four SARS-CoV-2 genes, Figure S3: Genomic distribution of nonsynonymous iSNVs in the ORF1a gene, Figure S4: Genomic distribution of nonsynonymous iSNVs in the ORF1b gene, Figure S5. iSNVs with distributions limited to specific Pangolin lineages, Table S1: iSNVs of 210 Australian samples, Table S2: Nucleotide substitutions observed in four SARS-CoV-2 genes from Australian samples, Table S3: Temporary groups of shared nonsynonymous iSNVs, Table S4: Correlation between the haplotype frequency and the alternative allele frequency, Table S5: Samples of PRJNA625551 BioProject presenting the haplotype N30G/S31Stop/F32Stop/R34P/G35R in the S gene; Table S6: Identification of the N30G/S31Stop/F32Stop/R34P/G35R haplotype with and without trimming ARTIC primers.
