# Supplementary material for: Intra-Host Diversity of SARS-Cov-2 Should Not Be Neglected: Case of the State of Victoria, Australia
Source: Viruses. 2021 Jan 19;13(1):133. doi: 10.3390/v13010133 (PMC7833370; doi:10.3390/v13010133)
Supplement: Supplementary file 1 [file viruses-13-00133-s001.pdf]

Supplementary Figure 1. Coverage and depth of 210 SARS-CoV-2 samples. The line represents the median value of the depth in each genomic position of four SARS-CoV-2 genes.

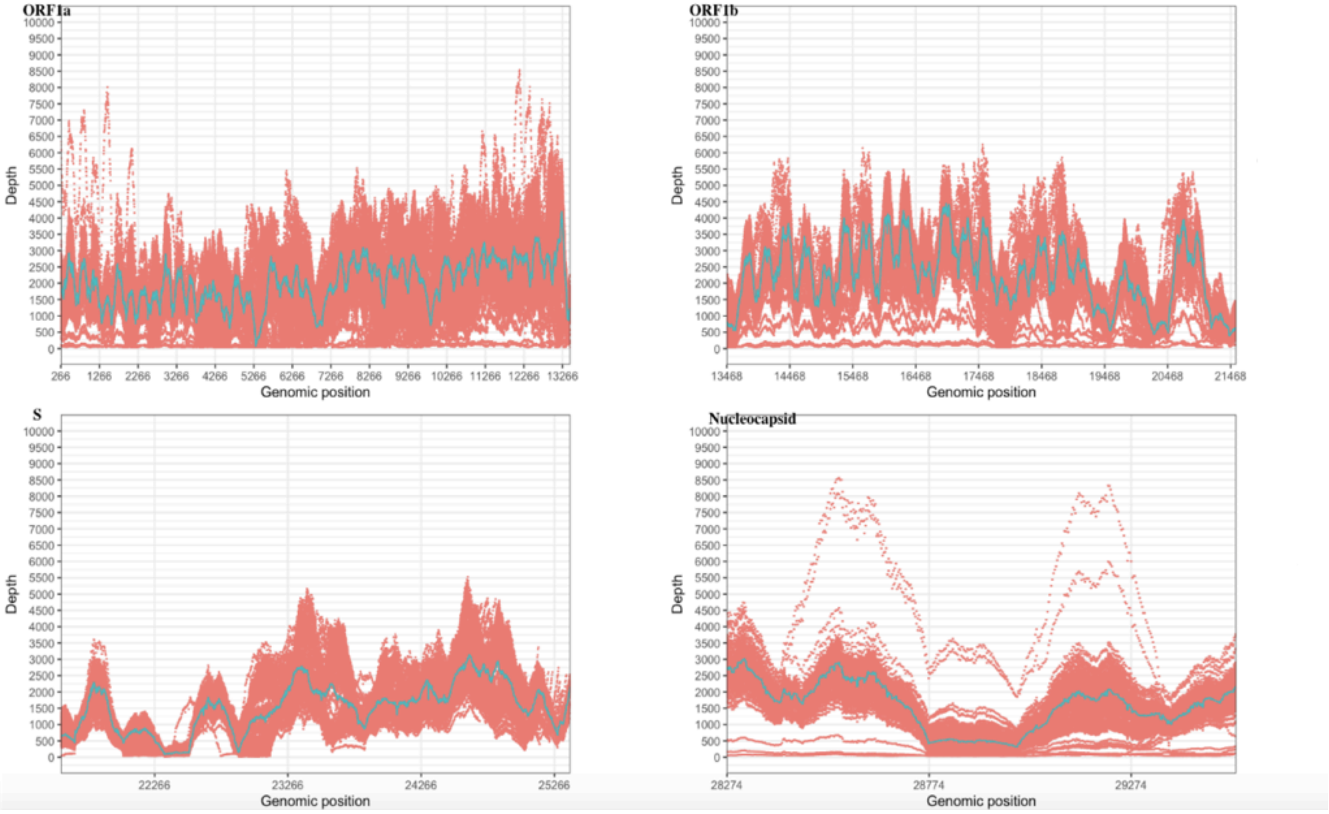

Supplementary Figure 2. Comparison of the density distributions of synonymous and nonsynonymous iSNVs in the four SARS-CoV-2 genes. The distributions of synonymous and nonsynonymous iSNVs were compared for the same sample with the Wilcoxon test.

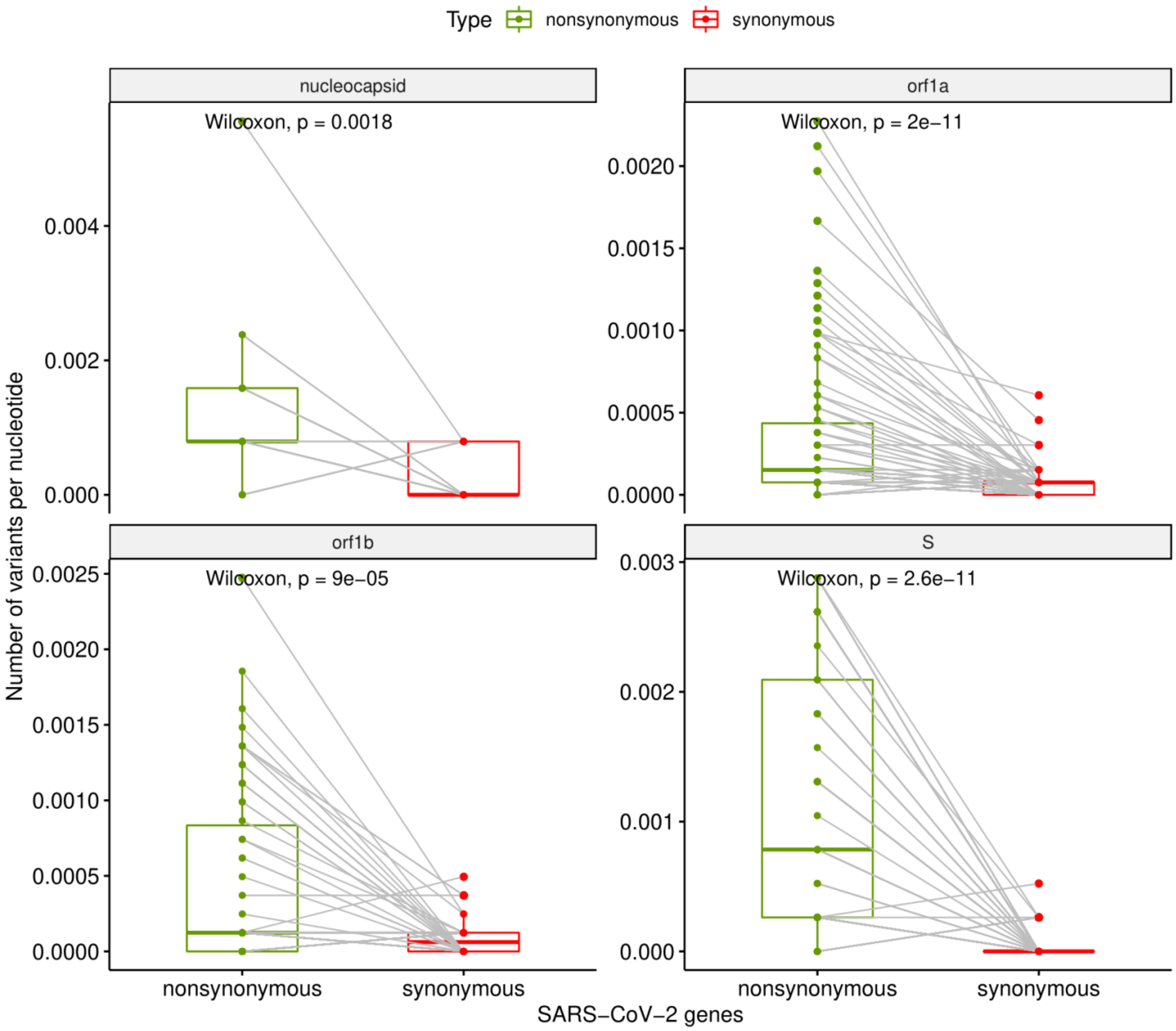

Supplementary Figure 3. Genomic distribution of nonsynonymous iSNVs in the ORF1a gene. Each dot represents the presence of a specific variant in a specific sample. On the vertical axis is represented the alternative allele frequency.

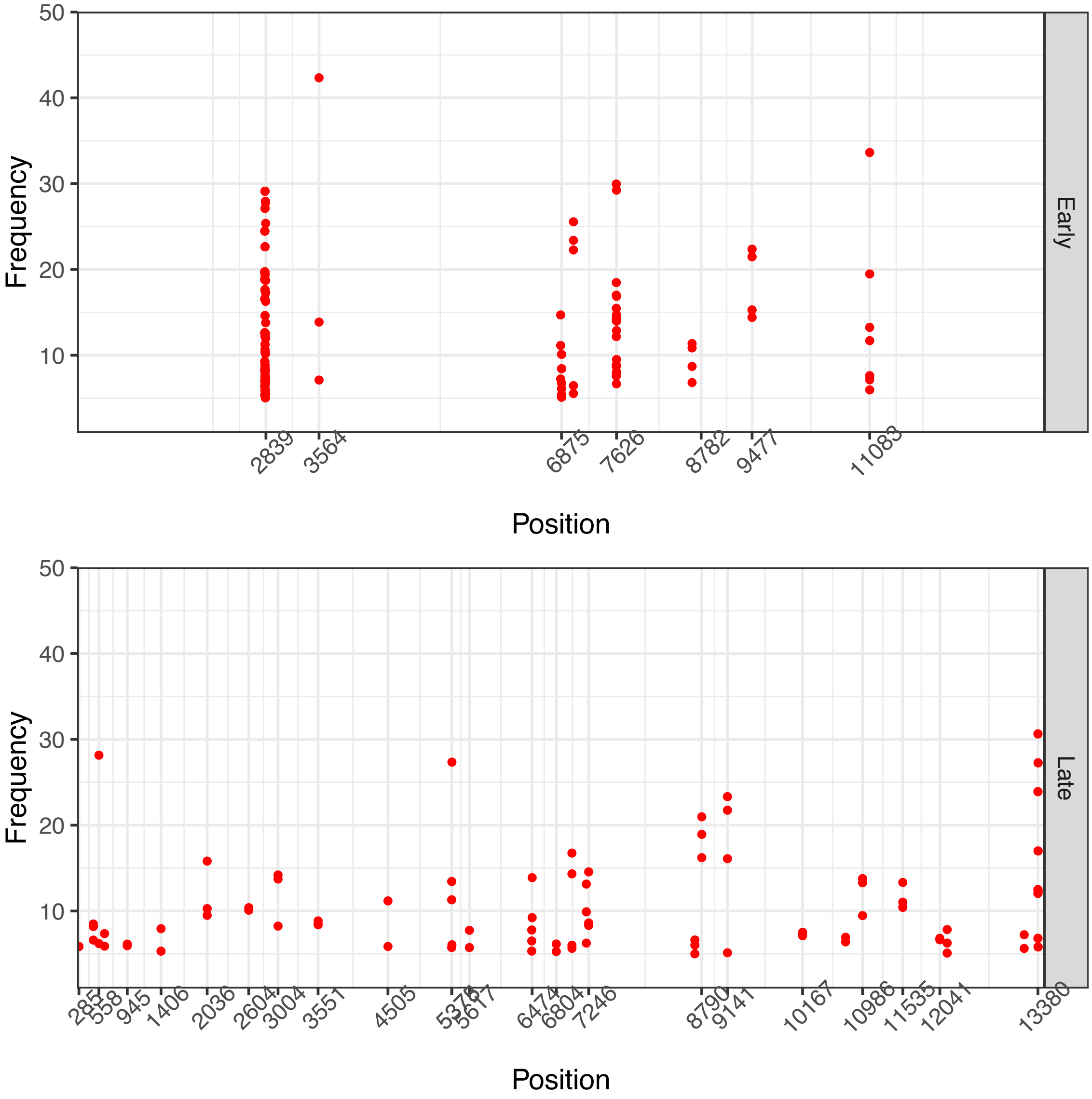

Supplementary Figure 4. Genomic distribution of nonsynonymous iSNVs in the ORF1b gene. Each dot represents the presence of a specific variant in a specific sample. On the vertical axis is represented the alternative allele frequency.

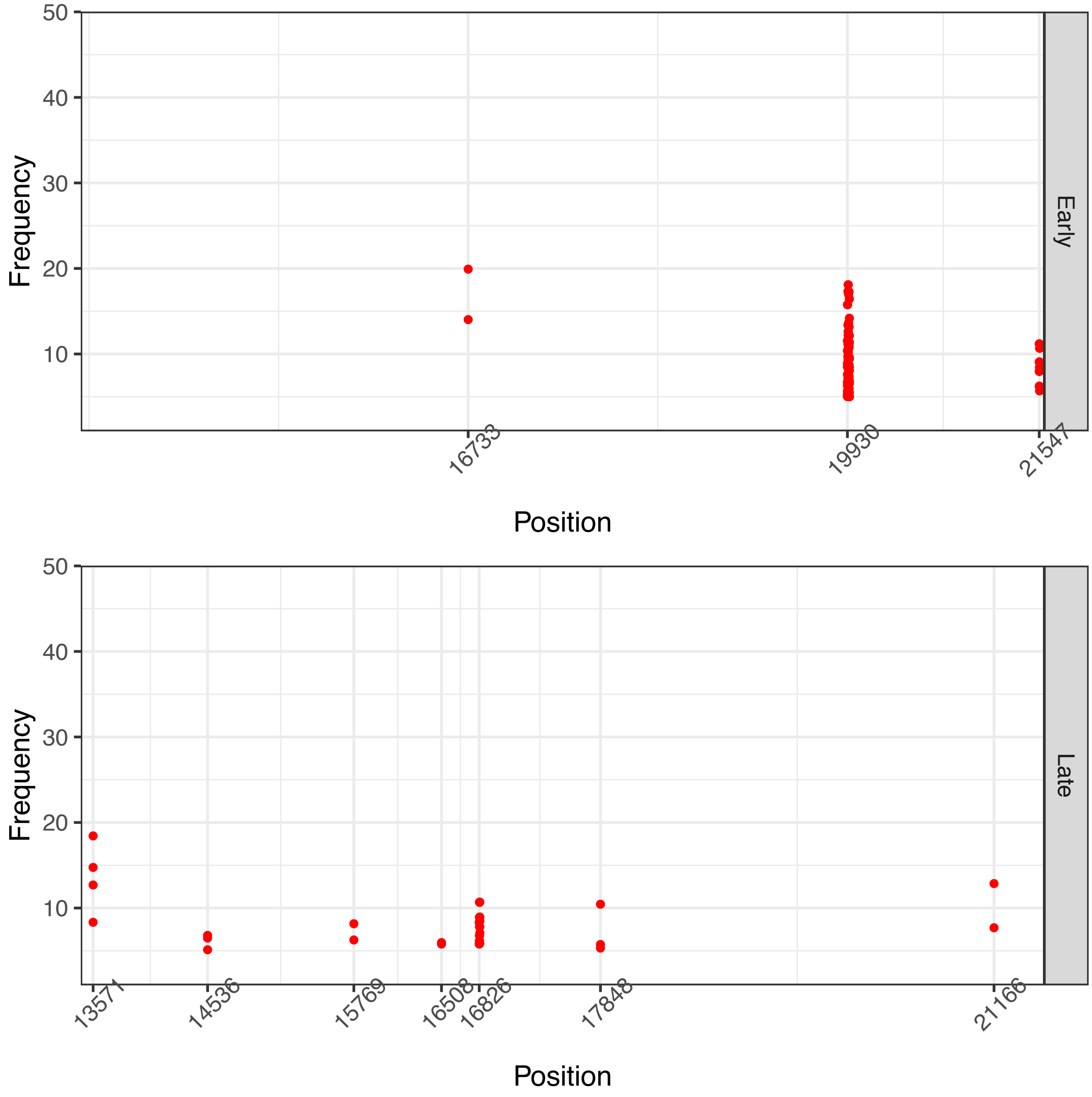

Supplementary Figure 5. iSNVs with distributions limited to specific Pangolin lineages. This heatmap presents the alternative allele frequency of iSNVs that were exclusive to D614/P314 or G614/L314 clades from the Early group. The Pangolin lineages are presented on the vertical axis. Some variants presented a specific distribution: for example, the T9223C, T9476A and T9477C ORF1a iSNVs were observed only in samples classified in the B.2.2 lineage.

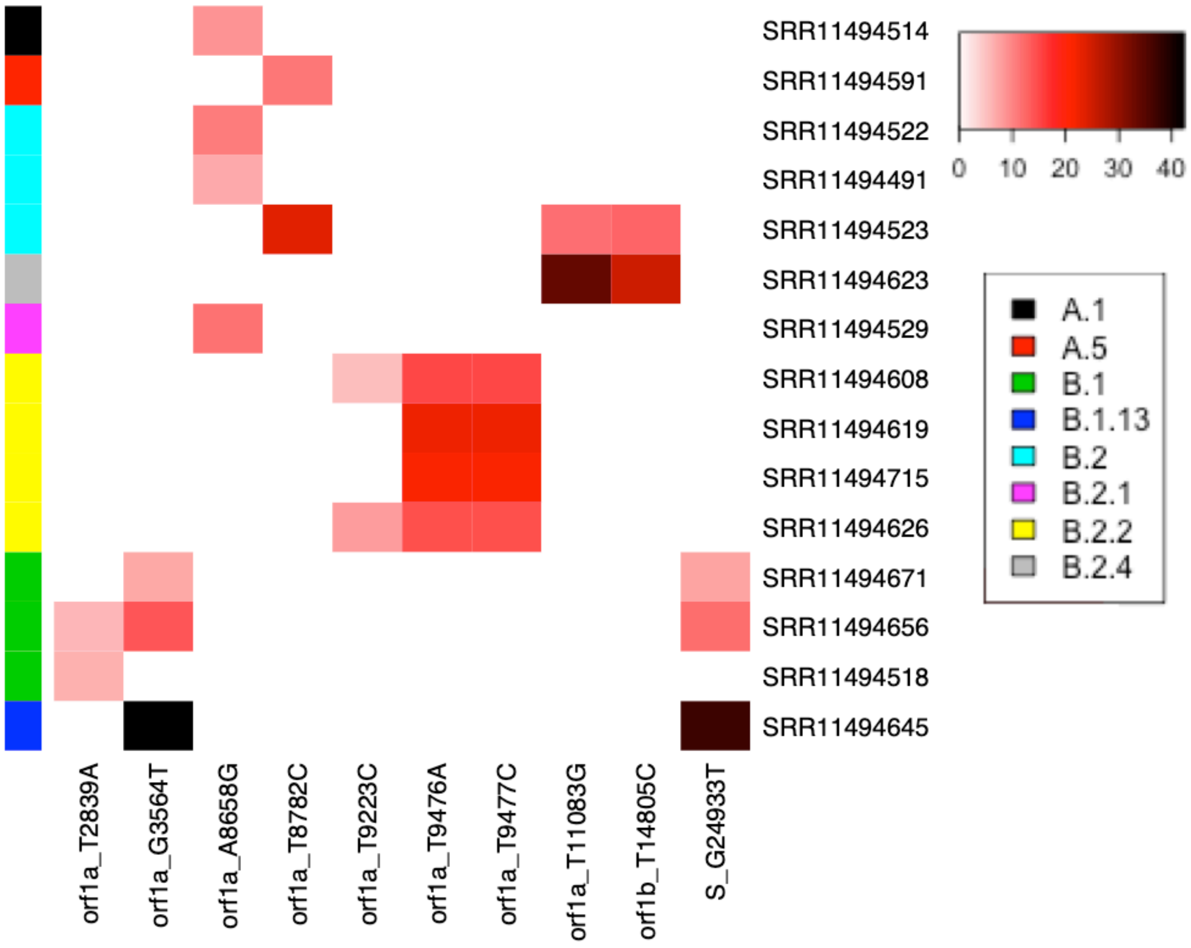

Supplementary Table 2. Nucleotide substitutions observed in the four SARS-CoV-2 genes from Australian samples. Nucleotide substitutions, number and frequency of iSNVs identified in patient samples from the state of Victoria, Australia.

Substitutions	Number of iSNVs	% of iSNVs
G>T	107	22
C>T	86	17
T>C	60	12
A>G	53	11
G>A	49	10
A>T	39	8
T>A	28	6
T>G	25	5
G>C	14	3
A>C	14	3
C>A	13	3
C>G	5	1

Supplementary Table 3. Temporary groups of shared nonsynonymous iSNVs. The Early group includes iSNVs that emerged in late January, while the Late group represents variants that were only observed after March 23. The proportions of samples bearing the respective variant are enclosed in parentheses.

	Early		Late	
	Synonymous	Nonsynonymous	Synonymous	Nonsynonymous
orf1a	T8782C(1.44),T9223C(0.96),T2839A(3.85)	T9476A(1.92),G2833C(5.29),T9477C(1.92),G2835T(4.81),A6863C(1.44),G3564T(1.44),A2825G(8.65),T11083G(0.96),G11083T(2.88),T2823A(5.29),A7626G(5.77),A2831C(5.29),A8658G(1.92),T2824G(5.29),A2826C(8.65),G6877T(2.88),T2827A(8.65),T7623G(4.33),C2822T(5.29),A6875T(2.88),G7038T(2.40)	G13240T(0.96)	G7214T(1.44),G4505T(0.96),G8696T(1.44),T13382G(1.92),C482T(1.44),G945T(0.96),G13193T(0.96),G5378T(0.96),T13380C(2.40),G10167T(0.96),G5375T(1.44),A6474T(0.96),G3004T(1.44),G10754T(0.96),G8790T(1.44),G10986T(1.44),G2604T(0.96),G7246T(1.44),G13383A(1.92),G5617T(0.96),G6804T(0.96),C635T(0.96),G12041T(0.96),G285T(0.96),C6468T(1.44),G1406T(0.96),C13381A(2.40),G11535T(1.44),G9141T(1.92),G2036T(1.44),G558T(0.96),C12141T(1.44),G7017T(1.92),C3551T(1.44)
orf1b	C17550T(0.97),T14805C(0.97)	C19944A(5.31),G19936C(7.25),C19934G(5.31),C21549T(1.93),A21551T(1.93),G19947C(6.28),T19940G(6.76),A21550C(1.93),A21548T(1.93),G19942A(5.31),A19930G(5.80),A21547G(1.93),G19932C(5.80),C16733T(0.97),C19938T(7.25)	A16824T(1.93)	A16831T(2.90),A21166T(0.97),C14536T(1.45),G16827A(0.97),T16828C(2.42),A16829T(2.42),G15769T(0.97),G17848T(1.45),G16508T(0.97),A16826T(0.97),G13571T(1.93)
S	NA	C21654A(12.44),A21650G(10.36),G21663C(15.54),C21658A(15.03),G21665C(25.91),T21655A(12.44),A21651G(10.36),G24933T(1.55),T21664A(15.54),T21657A(15.03),T21652G(10.36)	NA	G22899T(1.55),G23343T(1.04),G24557T(1.55)
Nucleocapsid	NA	NA	NA	G28559T(1.54)

Supplementary Table 4. Correlation of haplotype frequency and alternative allele frequency. For each of the haplotypes, the correlation between the haplotype frequency and the alternative allele frequency (AAF) of one of the variants was estimated (The variant used in the comparison is highlighted in bold). The number of samples corresponds to the samples where the haplotype had a frequency ≥ 5% and where the variant had also an AAF ≥ 5%.

Gene	Haplotype	Number of samples	Median haplotype frequency	Median AAF	Correlation between frequencies
S	N30G/S31Stop/F32Stop/R34P/G**35R**	47	6.2	6.31	0.86
orf1a	L853Stop/**N854A**/K856H	17	13.1	12.2	0.97
orf1b	E1120V/Y1121L/**T1122S**	6	7.99	8.16	0.98
orf1b	M2155V/T2156S/D2157H/**I2158R**/A2159T/K2160N	11	7.56	9.33	0.74

Supplementary Table 5. Samples of PRJNA625551 BioProject presenting the haplotype N30G/S31Stop/F32Stop/R34P/G35R in the S gene. For each of the samples, the total number of reads and the number of reads carrying the group of mutations is indicated.

Sample	Haplotype	Number of of reads with haplotype	Total number of reads	Frequency	Date of collection
SRR11772217	N30G/S31Stop/F32Stop/R34P/G35R	16	120	13.33	2020-04
SRR11772235	N30G/S31Stop/F32Stop/R34P/G35R	15	317	4.73	2020-04
SRR11772226	N30G/S31Stop/F32Stop/R34P/G35R	6	62	9.68	2020-04
SRR11772238	N30G/S31Stop/F32Stop/R34P/G35R	5	87	5.75	2020-04
SRR11652930	N30G/S31Stop/F32Stop/R34P/G35R	16	50	32.00	2020-04
SRR11563865	N30G/S31Stop/F32Stop/R34P/G35R	7	205	3.41	2020-03
SRR11652923	N30G/S31Stop/F32Stop/R34P/G35R	16	113	14.16	2020-04
SRR11563871	N30G/S31Stop/F32Stop/R34P/G35R	7	228	3.07	2020-03
SRR11563859	N30G/S31Stop/F32Stop/R34P/G35R	9	171	5.26	2020-03
SRR11772224	N30G/S31Stop/F32Stop/R34P/G35R	9	196	4.59	2020-04

Supplementary Table 6. Identification of the N30G/S31Stop/F32Stop/R34P/G35R haplotype with and without trimming ARTIC primers. The haplotype N30G/S31Stop/F32Stop/R34P/G35R was identified with and without removing the primers of reads with the ivar tool [67] in the Australian dataset.

	Without ivar tool			With ivar tool		
Sample	Number of reads with haplotype	Total number of reads	Frequency	Number of reads with haplotype	Total number of reads	Frequency
SRR11494580	18	260	6.92	22	330	6.67
SRR11494603	18	261	6.90	26	331	7.85
SRR11494610	24	293	8.19	32	371	8.63
SRR11494616	27	527	5.12	34	766	4.44
SRR11494585	23	256	8.98	31	321	9.66
SRR11494708	19	332	5.72	25	455	5.49
SRR11494565	16	278	5.76	20	371	5.39
SRR11494590	12	208	5.77	14	249	5.62
SRR11494459	24	301	7.97	29	375	7.73
SRR11494555	20	319	6.27	30	403	7.44
SRR11494548	22	290	7.59	28	377	7.43
SRR11494727	30	385	7.79	44	524	8.40
SRR11494530	16	258	6.20	23	334	6.89
SRR11494588	22	288	7.64	25	364	6.87
SRR11494486	23	270	8.52	29	349	8.31
SRR11494490	17	251	6.77	21	312	6.73
SRR11494605	15	287	5.23	17	373	4.56
SRR11494679	23	403	5.71	33	522	6.32
SRR11494615	16	309	5.18	28	420	6.67
SRR11494618	19	269	7.06	23	344	6.69
SRR11494600	25	358	6.98	36	489	7.36
SRR11494609	21	304	6.91	22	405	5.43
SRR11494576	19	376	5.05	25	495	5.05

SRR11494560	21	391	5.37	26	557	4.67
SRR11494519	24	336	7.14	32	436	7.34
SRR11494617	19	336	5.65	25	440	5.68
SRR11494568	24	319	7.52	32	424	7.55
SRR11494592	20	349	5.73	22	439	5.01
SRR11494537	19	226	8.41	27	266	10.15
SRR11494591	21	344	6.10	28	460	6.09
SRR11494567	21	327	6.42	29	432	6.71
SRR11494683	24	341	7.04	26	449	5.79
SRR11494751	24	366	6.56	32	483	6.63
SRR11494587	21	366	5.74	30	504	5.95
SRR11494562	21	382	5.50	29	514	5.64
SRR11494581	20	329	6.08	24	446	5.38
SRR11494455	21	345	6.09	27	450	6.00
SRR11494566	21	224	9.38	29	292	9.93
SRR11494465	20	300	6.67	22	371	5.93
SRR11494589	21	415	5.06	21	574	3.66
SRR11494559	21	297	7.07	25	352	7.10
SRR11494607	23	362	6.35	26	484	5.37
SRR11494612	18	306	5.88	26	413	6.30
SRR11494621	17	301	5.65	24	382	6.28
SRR11494569	19	350	5.43	25	473	5.29
SRR11494578	31	295	10.51	41	386	10.62
SRR11494582	20	398	5.03	27	564	4.79

Supplementary Table 1. iSNVs of 210 Australian samples.
The table presents the main information of iSNVs identified in the genes ORF1a, ORF1b, Spike (S) and nucleocapsid (N). The alternative allele frequency (AAF) was estimated with VirVarSeq and V-Phaser2 software.

Sample	Gene	Type	Nucleotide substitution	Aminoacid substitution	AAF VirVarSeq	AAF V-Phaser2	Data collection
SRR11494585	orf1a	nonsynonymous	T7623G	V2453G	8.84	1.46	3/13/2020
SRR11494585	orf1a	nonsynonymous	G11146T	M3627I	5.43	5.96	3/13/2020
SRR11494585	orf1a	nonsynonymous	A8732T	T2823S	5.52	6.76	3/13/2020
SRR11494585	orf1a	nonsynonymous	A7626G	N2454S	8.06	7.12	3/13/2020
SRR11494585	orf1a	nonsynonymous	G1440A	G392D	7.76	6.57	3/13/2020
SRR11494585	orf1a	nonsynonymous	G2835T	C857F	18.71	9.83	3/13/2020
SRR11494585	orf1a	nonsynonymous	T1059C	T265T	7.26	7.70	3/13/2020
SRR11494585	orf1a	nonsynonymous	G12988T	M4241I	7.65	8.07	3/13/2020
SRR11494585	orf1a	nonsynonymous	C7011T	A2249V	20.97	19.62	3/13/2020
SRR11494585	orf1a	nonsynonymous	G9312T	G3016V	5.34	4.70	3/13/2020
SRR11494585	orf1a	synonymous	G1855T	L530	7.42	6.20	3/13/2020
SRR11494585	orf1a	nonsynonymous	C10232T	R3323C	7.12	6.69	3/13/2020
SRR11494585	orf1a	nonsynonymous	G3692T	V1143F	9.99	9.88	3/13/2020
SRR11494585	orf1a	nonsynonymous	A2825G	N854A	29.11	25.26	3/13/2020
SRR11494585	orf1a	nonsynonymous	T2827A	N854A	29.11	26.32	3/13/2020
SRR11494585	orf1a	nonsynonymous	A2826C	N854A	29.11	25.53	3/13/2020
SRR11494645	orf1a	nonsynonymous	C12141T	T3959I	5.10	5.35	3/23/2020
SRR11494645	orf1a	nonsynonymous	G7214T	D2317Y	9.90	8.84	3/23/2020
SRR11494645	orf1a	nonsynonymous	G7038T	G2258V	22.27	21.69	3/23/2020
SRR11494645	orf1a	nonsynonymous	G2604T	G780V	10.39	9.13	3/23/2020
SRR11494645	orf1a	nonsynonymous	G285T	G7V	5.86	5.25	3/23/2020
SRR11494645	orf1a	nonsynonymous	G3004T	E913D	13.73	13.65	3/23/2020
SRR11494645	orf1a	nonsynonymous	G5375T	A1704S	5.74	5.22	3/23/2020
SRR11494645	orf1a	nonsynonymous	G945T	G227V	6.14	5.68	3/23/2020
SRR11494645	orf1a	nonsynonymous	G9141T	G2959V	23.33	23.55	3/23/2020
SRR11494645	orf1a	nonsynonymous	G8696T	A2811S	6.03	5.91	3/23/2020
SRR11494645	orf1a	nonsynonymous	C482T	R73C	8.20	8.43	3/23/2020
SRR11494645	orf1a	nonsynonymous	G10167T	C3301F	7.52	6.27	3/23/2020
SRR11494645	orf1a	nonsynonymous	G4505T	G1414C	5.87	4.66	3/23/2020
SRR11494645	orf1a	synonymous	A12274G	K4003	13.35	13.05	3/23/2020
SRR11494645	orf1a	nonsynonymous	G558T	G98V	28.16	27.14	3/23/2020
SRR11494645	orf1a	nonsynonymous	G8790T	G2842V	18.94	19.40	3/23/2020
SRR11494645	orf1a	nonsynonymous	G10754T	A3497S	6.40	5.15	3/23/2020
SRR11494645	orf1a	nonsynonymous	G3564T	G1100V	42.33	41.87	3/23/2020
SRR11494645	orf1a	nonsynonymous	G12041T	D3926Y	6.65	6.42	3/23/2020
SRR11494645	orf1a	nonsynonymous	G2036T	A591S	9.50	9.49	3/23/2020
SRR11494645	orf1a	nonsynonymous	G10986T	R3574I	9.47	9.43	3/23/2020
SRR11494645	orf1a	nonsynonymous	G5617T	Q1784H	5.74	7.01	3/23/2020
SRR11494645	orf1a	nonsynonymous	G11535T	G3757V	10.42	10.51	3/23/2020

SRR11494645	orf1a	nonsynonymous	G7017T	G2251V	14.34	14.63	3/23/2020
SRR11494645	orf1a	nonsynonymous	G7246T	W2327C	8.32	8.98	3/23/2020
SRR11494645	orf1a	nonsynonymous	C3551T	P1096S	8.46	8.33	3/23/2020
SRR11494645	orf1a	nonsynonymous	C6468T	T2068I	6.51	6.44	3/23/2020
SRR11494559	orf1a	synonymous	T3037C	F924	6.92	7.66	3/15/2020
SRR11494559	orf1a	nonsynonymous	A1515G	H417R	9.08	9.76	3/15/2020
SRR11494559	orf1a	nonsynonymous	G11083T	L3606F	7.62	7.96	3/15/2020
SRR11494520	orf1a	nonsynonymous	G1820A	G519S	5.97	5.52	3/17/2020
SRR11494685	orf1a	synonymous	T12748C	T4161	22.00	21.86	3/22/2020
SRR11494685	orf1a	nonsynonymous	G6877T	K2204Y	10.10	20.37	3/22/2020
SRR11494685	orf1a	nonsynonymous	A6875T	K2204Y	10.10	7.14	3/22/2020
SRR11494493	orf1a	nonsynonymous	G2835T	C857F	5.50	4.64	3/18/2020
SRR11494493	orf1a	nonsynonymous	T2823A	L853Stop	5.37	4.48	3/18/2020
SRR11494493	orf1a	nonsynonymous	C2822T	L853Stop	5.37	4.36	3/18/2020
SRR11494493	orf1a	nonsynonymous	T2824G	L853Stop	5.37	5.58	3/18/2020
SRR11494591	orf1a	synonymous	T8782C	S2839	10.98	11.13	3/13/2020
SRR11494591	orf1a	nonsynonymous	A7007G	A2248A	10.19	10.78	3/13/2020
SRR11494591	orf1a	nonsynonymous	G11083T	L3606F	7.58	9.34	3/13/2020
SRR11494591	orf1a	nonsynonymous	A6875T	K2204Y	5.38	2.22	3/13/2020
SRR11494591	orf1a	nonsynonymous	G6877T	K2204Y	5.38	8.03	3/13/2020
SRR11578322	orf1a	nonsynonymous	A2825G	N854A	10.41	7.64	3/28/2020
SRR11578322	orf1a	nonsynonymous	T2827A	N854A	10.41	7.75	3/28/2020
SRR11578322	orf1a	nonsynonymous	A2826C	N854A	10.41	7.69	3/28/2020
SRR11578322	orf1a	nonsynonymous	A7626G	N2454S	12.87	11.02	3/28/2020
SRR11578322	orf1a	nonsynonymous	C635T	R124C	7.36	6.48	3/28/2020
SRR11578322	orf1a	nonsynonymous	T7623G	V2453G	12.17	2.39	3/28/2020
SRR11578178	orf1a	nonsynonymous	A7626G	N2454S	13.99	14.20	3/25/2020
SRR11578178	orf1a	nonsynonymous	A2826C	N854A	8.52	7.17	3/25/2020
SRR11578178	orf1a	nonsynonymous	A2825G	N854A	8.52	7.09	3/25/2020
SRR11578178	orf1a	nonsynonymous	T2827A	N854A	8.52	7.22	3/25/2020
SRR11578178	orf1a	nonsynonymous	T2824G	L853Stop	8.26	9.89	3/25/2020
SRR11578178	orf1a	nonsynonymous	T2823A	L853Stop	8.26	7.14	3/25/2020
SRR11578178	orf1a	nonsynonymous	C2822T	L853Stop	8.26	6.97	3/25/2020
SRR11494751	orf1a	synonymous	C11758T	P3831	10.44	11.92	03/09/2020
SRR11494491	orf1a	synonymous	T9697C	Y3144	7.91	8.48	3/18/2020
SRR11494491	orf1a	nonsynonymous	A8658G	K2798R	6.82	37.27	3/18/2020
SRR11494491	orf1a	nonsynonymous	G1068A	G268E	5.02	4.41	3/18/2020
SRR11494654	orf1a	nonsynonymous	C3885T	P1207L	8.81	9.08	03/10/2020
SRR11494654	orf1a	synonymous	C3874T	I1203	6.77	6.57	03/10/2020
SRR11494573	orf1a	synonymous	A10081G	P3272	44.02	45.18	3/14/2020
SRR11494762	orf1a	nonsynonymous	G7360A	M2365I	12.15	2.49	2/21/2020
SRR11494762	orf1a	nonsynonymous	A1814T	K517Stop	6.11	6.81	2/21/2020
SRR11494762	orf1a	nonsynonymous	A5769G	H1835R	5.60	5.16	2/21/2020
SRR11494762	orf1a	synonymous	T565C	S100	5.04	4.25	2/21/2020
SRR11494762	orf1a	nonsynonymous	G10281A	R3339K	6.93	6.66	2/21/2020
SRR11494762	orf1a	nonsynonymous	T10653A	I3463N	7.34	2.92	2/21/2020

SRR11494762	orf1a	nonsynonymous	T10654C	I3463N	7.34	2.74	2/21/2020
SRR11494762	orf1a	nonsynonymous	C10656A	T3464K	7.39	2.45	2/21/2020
SRR11494762	orf1a	nonsynonymous	T7364G	L2367V	12.32	2.64	2/21/2020
SRR11494762	orf1a	synonymous	T5080G	T1605	7.23	6.98	2/21/2020
SRR11494762	orf1a	nonsynonymous	C6696T	P2144L	6.14	6.42	2/21/2020
SRR11494762	orf1a	nonsynonymous	T9423C	V3053A	7.16	11.49	2/21/2020
SRR11494762	orf1a	nonsynonymous	C8262T	T2666I	11.39	0.47	2/21/2020
SRR11494762	orf1a	nonsynonymous	T8263A	T2666I	11.39	0.50	2/21/2020
SRR11494762	orf1a	synonymous	T10660C	V3465	8.00	1.16	2/21/2020
SRR11494762	orf1a	synonymous	C11737T	N3824	33.95	33.37	2/21/2020
SRR11494762	orf1a	nonsynonymous	A9026T	I2921F	5.15	5.38	2/21/2020
SRR11494574	orf1a	nonsynonymous	G2659T	K798N	6.42	7.16	3/14/2020
SRR11494565	orf1a	synonymous	A4952C	R1563	13.14	13.49	3/15/2020
SRR11494568	orf1a	nonsynonymous	G1515A	H417H	40.95	39.58	3/14/2020
SRR11494555	orf1a	nonsynonymous	G2833C	K856H	5.03	4.12	3/16/2020
SRR11494555	orf1a	nonsynonymous	A2831C	K856H	5.03	5.25	3/16/2020
SRR11622002	orf1a	nonsynonymous	A7626G	N2454S	14.32	16.70	04/03/2020
SRR11622002	orf1a	nonsynonymous	T2824G	L853Stop	9.27	9.65	04/03/2020
SRR11622002	orf1a	nonsynonymous	T2823A	L853Stop	9.27	7.04	04/03/2020
SRR11622002	orf1a	nonsynonymous	C2822T	L853Stop	9.27	6.76	04/03/2020
SRR11622002	orf1a	nonsynonymous	A2826C	N854A	8.84	6.95	04/03/2020
SRR11622002	orf1a	nonsynonymous	A2825G	N854A	8.84	6.84	04/03/2020
SRR11622002	orf1a	nonsynonymous	T2827A	N854A	8.84	7.21	04/03/2020
SRR11622002	orf1a	nonsynonymous	T7623G	V2453G	14.32	2.63	04/03/2020
SRR11622002	orf1a	nonsynonymous	G13383A	C4373D	12.31	9.34	04/03/2020
SRR11622002	orf1a	nonsynonymous	T13382G	C4373D	12.31	6.57	04/03/2020
SRR11622002	orf1a	nonsynonymous	C13381A	V4372A	12.06	6.86	04/03/2020
SRR11622002	orf1a	nonsynonymous	T13380C	V4372A	12.06	5.69	04/03/2020
SRR11578323	orf1a	nonsynonymous	A7626G	N2454S	13.99	15.34	3/28/2020
SRR11578318	orf1a	nonsynonymous	G5634T	G1790V	7.14	4.46	3/27/2020
SRR11578318	orf1a	nonsynonymous	G8591A	V2776I	8.16	8.05	3/27/2020
SRR11578318	orf1a	nonsynonymous	C13381A	V4372A	6.82	3.85	3/27/2020
SRR11578318	orf1a	nonsynonymous	T13380C	V4372A	6.82	3.77	3/27/2020
SRR11578318	orf1a	nonsynonymous	T13382G	C4373D	5.81	3.85	3/27/2020
SRR11578318	orf1a	nonsynonymous	G13383A	C4373D	5.81	3.85	3/27/2020
SRR11578318	orf1a	nonsynonymous	T7623G	V2453G	8.71	1.77	3/27/2020
SRR11578318	orf1a	nonsynonymous	A7626G	N2454S	7.93	6.11	3/27/2020
SRR11494554	orf1a	nonsynonymous	G4683C	A1473A	6.92	2.31	3/16/2020
SRR11621993	orf1a	nonsynonymous	T7623G	V2453G	29.96	5.43	04/01/2020
SRR11621993	orf1a	nonsynonymous	A6474T	E2070V	13.89	9.50	04/01/2020
SRR11621993	orf1a	nonsynonymous	C2822T	L853Stop	12.62	6.86	04/01/2020
SRR11621993	orf1a	nonsynonymous	T2823A	L853Stop	12.62	6.64	04/01/2020
SRR11621993	orf1a	nonsynonymous	T2824G	L853Stop	12.62	13.82	04/01/2020
SRR11621993	orf1a	nonsynonymous	A2826C	N854A	11.27	6.57	04/01/2020
SRR11621993	orf1a	nonsynonymous	A2825G	N854A	11.27	7.33	04/01/2020
SRR11621993	orf1a	nonsynonymous	T2827A	N854A	11.27	6.48	04/01/2020

SRR11621993	orf1a	nonsynonymous	C5866G	F1867V	9.68	16.67	04/01/2020
SRR11621993	orf1a	nonsynonymous	T5864G	F1867V	9.68	14.29	04/01/2020
SRR11621993	orf1a	nonsynonymous	A7626G	N2454S	29.24	35.09	04/01/2020
SRR11494497	orf1a	synonymous	C11747T	L3828	15.11	16.22	3/17/2020
SRR11494497	orf1a	nonsynonymous	G581A	V106I	7.77	7.19	3/17/2020
SRR11494523	orf1a	synonymous	T8782C	S2839	23.40	25.00	3/17/2020
SRR11494523	orf1a	synonymous	C3253T	D996	7.62	7.71	3/17/2020
SRR11494523	orf1a	synonymous	C10582T	D3439	8.79	9.11	3/17/2020
SRR11494523	orf1a	nonsynonymous	A1460G	K399E	10.47	10.68	3/17/2020
SRR11494523	orf1a	synonymous	T1090C	F275	5.24	5.62	3/17/2020
SRR11494523	orf1a	nonsynonymous	T9464C	F3067L	5.37	5.41	3/17/2020
SRR11494523	orf1a	nonsynonymous	A12381G	K4039R	6.81	6.53	3/17/2020
SRR11494523	orf1a	nonsynonymous	T11083G	L3606L	11.70	12.22	3/17/2020
SRR11621987	orf1a	nonsynonymous	A9146G	I2961V	6.93	6.90	04/01/2020
SRR11621987	orf1a	nonsynonymous	G7626A	N2454N	44.35	49.21	04/01/2020
SRR11621987	orf1a	nonsynonymous	A5538T	Q1758L	6.99	7.53	04/01/2020
SRR11621987	orf1a	nonsynonymous	A6716C	S2151R	5.69	5.42	04/01/2020
SRR11621987	orf1a	nonsynonymous	T1791G	F509C	5.13	4.88	04/01/2020
SRR11621987	orf1a	nonsynonymous	T13382G	C4373D	27.27	13.64	04/01/2020
SRR11621987	orf1a	nonsynonymous	G13383A	C4373D	27.27	16.67	04/01/2020
SRR11621987	orf1a	nonsynonymous	G10523C	V3420L	8.10	8.03	04/01/2020
SRR11621987	orf1a	nonsynonymous	G11266T	L3667F	7.46	8.21	04/01/2020
SRR11621987	orf1a	nonsynonymous	C13381A	V4372A	23.91	11.43	04/01/2020
SRR11621987	orf1a	nonsynonymous	T13380C	V4372A	23.91	10.67	04/01/2020
SRR11621987	orf1a	nonsynonymous	T7218A	L2318Stop	6.52	6.17	04/01/2020
SRR11621987	orf1a	nonsynonymous	G2835T	C857F	12.50	6.47	04/01/2020
SRR11494588	orf1a	nonsynonymous	A2826C	N854A	5.89	3.63	3/13/2020
SRR11494588	orf1a	nonsynonymous	A2825G	N854A	5.89	3.61	3/13/2020
SRR11494588	orf1a	nonsynonymous	T2827A	N854A	5.89	3.64	3/13/2020
SRR11494588	orf1a	nonsynonymous	G2833C	K856H	5.71	4.90	3/13/2020
SRR11494588	orf1a	nonsynonymous	A2831C	K856H	5.71	6.10	3/13/2020
SRR11494588	orf1a	nonsynonymous	C2822T	L853Stop	6.38	3.53	3/13/2020
SRR11494588	orf1a	nonsynonymous	T2823A	L853Stop	6.38	3.62	3/13/2020
SRR11494588	orf1a	nonsynonymous	T2824G	L853Stop	6.38	5.54	3/13/2020
SRR11578325	orf1a	nonsynonymous	T9206A	S2981T	6.32	1.15	3/28/2020
SRR11578325	orf1a	nonsynonymous	A9175C	E2970D	15.76	15.63	3/28/2020
SRR11494519	orf1a	nonsynonymous	G11083T	L3606F	13.25	15.71	3/16/2020
SRR11494519	orf1a	nonsynonymous	A2825G	N854A	12.21	10.09	3/16/2020
SRR11494519	orf1a	nonsynonymous	T2827A	N854A	12.21	10.55	3/16/2020
SRR11494519	orf1a	nonsynonymous	A2826C	N854A	12.21	10.60	3/16/2020
SRR11494603	orf1a	nonsynonymous	A2831C	K856H	7.17	9.41	03/12/2020
SRR11494603	orf1a	nonsynonymous	G2833C	K856H	7.17	6.87	03/12/2020
SRR11494603	orf1a	nonsynonymous	A6875T	K2204Y	6.75	3.16	03/12/2020
SRR11494603	orf1a	nonsynonymous	G6877T	K2204Y	6.75	9.12	03/12/2020
SRR11494603	orf1a	nonsynonymous	A2826C	N854A	7.51	5.47	03/12/2020
SRR11494603	orf1a	nonsynonymous	T2827A	N854A	7.51	5.21	03/12/2020

SRR11494603	orf1a	nonsynonymous	A2825G	N854A	7.51	5.48	03/12/2020
SRR11494508	orf1a	synonymous	C11074T	F3603	7.87	12.45	3/16/2020
SRR11494508	orf1a	nonsynonymous	A2826C	N854A	7.97	4.55	3/16/2020
SRR11494508	orf1a	nonsynonymous	A2825G	N854A	7.97	5.01	3/16/2020
SRR11494508	orf1a	nonsynonymous	T2827A	N854A	7.97	4.84	3/16/2020
SRR11494508	orf1a	nonsynonymous	A2831C	K856H	7.48	8.36	3/16/2020
SRR11494508	orf1a	nonsynonymous	G2833C	K856H	7.48	6.42	3/16/2020
SRR11494727	orf1a	nonsynonymous	A2831C	K856H	8.24	10.85	03/09/2020
SRR11494727	orf1a	nonsynonymous	G2833C	K856H	8.24	8.53	03/09/2020
SRR11622072	orf1a	nonsynonymous	C4055A	H1264N	9.52	8.94	04/07/2020
SRR11622072	orf1a	nonsynonymous	T9727A	Y3154Stop	12.45	12.55	04/07/2020
SRR11622072	orf1a	nonsynonymous	A6474T	E2070V	9.23	9.37	04/07/2020
SRR11622072	orf1a	nonsynonymous	A4060G	P1265L	8.59	7.32	04/07/2020
SRR11622072	orf1a	nonsynonymous	C4059T	P1265L	8.59	8.12	04/07/2020
SRR11622072	orf1a	nonsynonymous	T6866A	N2201N	25.64	34.42	04/07/2020
SRR11622072	orf1a	nonsynonymous	A5636T	K1791Y	6.15	5.68	04/07/2020
SRR11622072	orf1a	nonsynonymous	A5638C	K1791Y	6.15	5.30	04/07/2020
SRR11622072	orf1a	nonsynonymous	G2809T	R848S	23.48	20.95	04/07/2020
SRR11622072	orf1a	nonsynonymous	A2730G	K822R	13.64	13.30	04/07/2020
SRR11622072	orf1a	nonsynonymous	T4054G	L1263Q	10.45	13.50	04/07/2020
SRR11622072	orf1a	nonsynonymous	T4053A	L1263Q	10.45	9.76	04/07/2020
SRR11622072	orf1a	nonsynonymous	A6863C	K2200Q	14.70	4.65	04/07/2020
SRR11622072	orf1a	nonsynonymous	A2826C	N854A	19.59	14.10	04/07/2020
SRR11622072	orf1a	nonsynonymous	A2825G	N854A	19.59	15.12	04/07/2020
SRR11622072	orf1a	nonsynonymous	T2827A	N854A	19.59	14.69	04/07/2020
SRR11622072	orf1a	synonymous	A5650G	K1795	5.15	2.61	04/07/2020
SRR11622072	orf1a	nonsynonymous	T4064G	S1267A	5.17	3.91	04/07/2020
SRR11622072	orf1a	nonsynonymous	G8371T	Q2702H	19.34	19.02	04/07/2020
SRR11622072	orf1a	synonymous	T2839A	S858	7.18	3.84	04/07/2020
SRR11494708	orf1a	nonsynonymous	C1392T	S376L	5.38	5.08	3/18/2020
SRR11494514	orf1a	nonsynonymous	A8658G	K2798R	8.70	29.34	3/18/2020
SRR11494514	orf1a	nonsynonymous	G5950T	K1895N	6.76	6.85	3/18/2020
SRR11494614	orf1a	synonymous	A4951G	L1562	5.32	5.23	03/11/2020
SRR11494469	orf1a	nonsynonymous	C12213T	S3983F	5.91	8.08	3/19/2020
SRR11494626	orf1a	synonymous	C2773T	Y836	5.23	6.58	3/24/2020
SRR11494626	orf1a	nonsynonymous	T9477C	F3071T	14.41	16.22	3/24/2020
SRR11494626	orf1a	nonsynonymous	T9476A	F3071T	14.41	18.40	3/24/2020
SRR11494626	orf1a	synonymous	T9223C	H2986	8.06	5.90	3/24/2020
SRR11494489	orf1a	synonymous	C4582T	N1439	6.33	5.57	3/19/2020
SRR11494489	orf1a	nonsynonymous	G3216T	S984I	17.06	18.39	3/19/2020
SRR11494459	orf1a	nonsynonymous	A6821G	T2186A	5.70	6.03	3/19/2020
SRR11494459	orf1a	nonsynonymous	G11083T	L3606F	5.97	6.36	3/19/2020
SRR11494550	orf1a	nonsynonymous	G1748A	D495N	15.53	15.58	3/16/2020
SRR11494550	orf1a	synonymous	T3370C	T1035	13.15	12.30	3/16/2020
SRR11494606	orf1a	nonsynonymous	T9129C	V2955A	19.75	20.07	03/11/2020
SRR11494606	orf1a	nonsynonymous	G8111A	A2616T	23.71	22.94	03/11/2020

SRR11494606	orf1a	nonsynonymous	G7553A	V2430I	21.46	20.76	03/11/2020
SRR11494662	orf1a	nonsynonymous	G2833C	K856H	10.19	10.33	3/23/2020
SRR11494662	orf1a	nonsynonymous	A2831C	K856H	10.19	16.03	3/23/2020
SRR11494662	orf1a	nonsynonymous	C5768A	H1835N	5.25	4.78	3/23/2020
SRR11494671	orf1a	nonsynonymous	G7038T	G2258V	5.54	4.68	3/22/2020
SRR11494671	orf1a	nonsynonymous	G3564T	G1100V	7.11	6.76	3/22/2020
SRR11494610	orf1a	nonsynonymous	G2833C	K856H	6.75	7.61	03/11/2020
SRR11494610	orf1a	nonsynonymous	A2831C	K856H	6.75	8.17	03/11/2020
SRR11494522	orf1a	nonsynonymous	T1107C	I281T	10.33	10.80	3/17/2020
SRR11494522	orf1a	nonsynonymous	A8658G	K2798R	10.85	41.00	3/17/2020
SRR11494557	orf1a	nonsynonymous	G10494T	S3410I	5.62	5.53	3/15/2020
SRR11494557	orf1a	synonymous	A5872G	K1869	6.19	5.04	3/15/2020
SRR11494557	orf1a	nonsynonymous	G3114A	G950D	5.48	5.06	3/15/2020
SRR11494557	orf1a	nonsynonymous	T7623G	V2453G	17.00	5.39	3/15/2020
SRR11494557	orf1a	nonsynonymous	G5861T	V1866F	8.31	7.15	3/15/2020
SRR11494557	orf1a	synonymous	C7594T	G2443	9.68	10.96	3/15/2020
SRR11494557	orf1a	synonymous	C9803T	L3180	6.52	5.20	3/15/2020
SRR11494557	orf1a	synonymous	A10172C	R3303	6.90	7.92	3/15/2020
SRR11494557	orf1a	nonsynonymous	G9998A	G3245S	8.58	7.53	3/15/2020
SRR11494557	orf1a	nonsynonymous	G2118T	G618V	6.01	4.62	3/15/2020
SRR11494557	orf1a	synonymous	T9127C	Y2954	5.78	5.82	3/15/2020
SRR11494557	orf1a	nonsynonymous	A7626G	N2454S	16.87	16.67	3/15/2020
SRR11494557	orf1a	nonsynonymous	C737T	Q158Stop	5.24	4.50	3/15/2020
SRR11494557	orf1a	nonsynonymous	G545T	G94C	6.08	4.99	3/15/2020
SRR11494557	orf1a	synonymous	A12895G	E4210	5.84	5.16	3/15/2020
SRR11494557	orf1a	nonsynonymous	C13377T	T4371I	7.69	12.60	3/15/2020
SRR11494557	orf1a	nonsynonymous	T7205C	F2314L	7.76	8.48	3/15/2020
SRR11494557	orf1a	nonsynonymous	T11711G	S3816A	5.82	4.94	3/15/2020
SRR11494557	orf1a	synonymous	T7249C	F2328	10.24	9.22	3/15/2020
SRR11494557	orf1a	synonymous	T2839A	S858	7.99	4.26	3/15/2020
SRR11494557	orf1a	nonsynonymous	G7273T	R2336S	5.90	7.15	3/15/2020
SRR11494657	orf1a	nonsynonymous	G7038T	G2258V	23.40	21.05	3/23/2020
SRR11494657	orf1a	nonsynonymous	C12141T	T3959I	7.84	8.68	3/23/2020
SRR11494657	orf1a	nonsynonymous	G7214T	D2317Y	13.14	12.00	3/23/2020
SRR11494657	orf1a	nonsynonymous	G11693T	V3810F	6.40	5.90	3/23/2020
SRR11494657	orf1a	nonsynonymous	G2604T	G780V	10.10	8.98	3/23/2020
SRR11494657	orf1a	nonsynonymous	G13193T	V4310F	5.64	5.61	3/23/2020
SRR11494657	orf1a	nonsynonymous	C482T	R73C	6.61	6.88	3/23/2020
SRR11494657	orf1a	nonsynonymous	G8696T	A2811S	6.63	6.35	3/23/2020
SRR11494657	orf1a	nonsynonymous	G10167T	C3301F	7.11	6.09	3/23/2020
SRR11494657	orf1a	nonsynonymous	C7194T	T2310I	6.14	5.74	3/23/2020
SRR11494657	orf1a	nonsynonymous	G3004T	E913D	8.24	8.34	3/23/2020
SRR11494657	orf1a	nonsynonymous	G285T	G7V	5.88	5.67	3/23/2020
SRR11494657	orf1a	nonsynonymous	G9141T	G2959V	21.75	22.09	3/23/2020
SRR11494657	orf1a	nonsynonymous	G945T	G227V	5.95	5.66	3/23/2020
SRR11494657	orf1a	nonsynonymous	G5375T	A1704S	11.31	10.26	3/23/2020

SRR11494657	orf1a	nonsynonymous	G8790T	G2842V	20.98	20.50	3/23/2020
SRR11494657	orf1a	nonsynonymous	G2036T	A591S	15.82	15.59	3/23/2020
SRR11494657	orf1a	nonsynonymous	G12041T	D3926Y	6.82	6.31	3/23/2020
SRR11494657	orf1a	nonsynonymous	C9110T	R2949C	5.08	5.38	3/23/2020
SRR11494657	orf1a	nonsynonymous	G6804T	C2180F	6.15	5.74	3/23/2020
SRR11494657	orf1a	nonsynonymous	G5233A	W1656Stop	5.28	7.16	3/23/2020
SRR11494657	orf1a	nonsynonymous	C6468T	T2068I	7.79	7.73	3/23/2020
SRR11494657	orf1a	nonsynonymous	C3551T	P1096S	8.85	9.54	3/23/2020
SRR11494657	orf1a	nonsynonymous	G1406T	E381Stop	7.94	7.10	3/23/2020
SRR11494657	orf1a	nonsynonymous	G5617T	Q1784H	7.75	10.27	3/23/2020
SRR11494657	orf1a	nonsynonymous	G10986T	R3574I	13.78	12.97	3/23/2020
SRR11494657	orf1a	nonsynonymous	G5847T	G1861V	9.22	10.04	3/23/2020
SRR11494657	orf1a	nonsynonymous	G7246T	W2327C	8.61	9.36	3/23/2020
SRR11494657	orf1a	synonymous	G13240T	S4325	7.07	7.01	3/23/2020
SRR11494657	orf1a	nonsynonymous	G7017T	G2251V	16.75	17.93	3/23/2020
SRR11494657	orf1a	nonsynonymous	G11535T	G3757V	11.03	11.44	3/23/2020
SRR11578167	orf1a	nonsynonymous	C1457T	R398C	24.73	24.93	3/24/2020
SRR11494538	orf1a	synonymous	C4084T	D1273	36.20	36.96	3/15/2020
SRR11494655	orf1a	synonymous	G10870T	L3535	35.66	34.34	3/23/2020
SRR11494612	orf1a	synonymous	G343T	V26	5.94	5.14	03/11/2020
SRR11494615	orf1a	nonsynonymous	G2833C	K856H	5.78	5.53	03/11/2020
SRR11494615	orf1a	nonsynonymous	A2831C	K856H	5.78	6.94	03/11/2020
SRR11494687	orf1a	synonymous	C8140T	S2625	19.20	20.46	3/22/2020
SRR11494529	orf1a	nonsynonymous	A13467G	N4401S	6.09	5.22	3/17/2020
SRR11494529	orf1a	nonsynonymous	A8658G	K2798R	11.36	38.26	3/17/2020
SRR11494577	orf1a	nonsynonymous	T2827A	N854A	14.62	11.59	3/14/2020
SRR11494577	orf1a	nonsynonymous	A2825G	N854A	14.62	11.22	3/14/2020
SRR11494577	orf1a	nonsynonymous	A2826C	N854A	14.62	12.14	3/14/2020
SRR11494577	orf1a	nonsynonymous	T2824G	L853Stop	16.57	15.61	3/14/2020
SRR11494577	orf1a	nonsynonymous	T2823A	L853Stop	16.57	11.33	3/14/2020
SRR11494577	orf1a	nonsynonymous	C2822T	L853Stop	16.57	11.22	3/14/2020
SRR11494577	orf1a	synonymous	T2839A	S858	6.99	3.69	3/14/2020
SRR11494548	orf1a	nonsynonymous	C884T	R207C	6.54	7.48	1/31/2020
SRR11494623	orf1a	nonsynonymous	T3096C	S944S	25.08	24.80	3/24/2020
SRR11494623	orf1a	nonsynonymous	G4298T	V1345L	40.17	38.81	3/24/2020
SRR11494623	orf1a	nonsynonymous	T11083G	L3606L	33.63	28.11	3/24/2020
SRR11494623	orf1a	nonsynonymous	G1607A	D448N	9.98	1.86	3/24/2020
SRR11494587	orf1a	nonsynonymous	G2833C	K856H	5.93	5.78	3/13/2020
SRR11494587	orf1a	nonsynonymous	A2831C	K856H	5.93	6.60	3/13/2020
SRR11494715	orf1a	nonsynonymous	T9477C	F3071T	21.48	28.88	03/10/2020
SRR11494715	orf1a	nonsynonymous	T9476A	F3071T	21.48	29.41	03/10/2020
SRR11494551	orf1a	nonsynonymous	G6647A	A2128T	6.82	6.24	3/16/2020
SRR11494638	orf1a	nonsynonymous	G5378T	G1705C	27.35	4.59	3/23/2020
SRR11494594	orf1a	nonsynonymous	A6459G	N2065S	6.88	7.25	03/12/2020
SRR11494594	orf1a	nonsynonymous	G5927A	V1888I	9.46	13.05	03/12/2020
SRR11494594	orf1a	synonymous	G6793A	L2176	9.05	8.42	03/12/2020

SRR11494478	orf1a	nonsynonymous	G2231T	A656S	5.12	4.64	03/08/2020
SRR11494478	orf1a	synonymous	T2335C	A690	7.59	7.87	03/08/2020
SRR11494478	orf1a	nonsynonymous	A1935T	N557I	5.72	3.67	03/08/2020
SRR11494478	orf1a	nonsynonymous	A2826C	N854A	10.64	6.94	03/08/2020
SRR11494478	orf1a	nonsynonymous	T2827A	N854A	10.64	7.06	03/08/2020
SRR11494478	orf1a	nonsynonymous	A2825G	N854A	10.64	6.32	03/08/2020
SRR11494486	orf1a	nonsynonymous	T2824G	L853Stop	24.47	26.45	3/17/2020
SRR11494486	orf1a	nonsynonymous	C2822T	L853Stop	24.47	16.38	3/17/2020
SRR11494486	orf1a	nonsynonymous	T2823A	L853Stop	24.47	16.38	3/17/2020
SRR11494486	orf1a	synonymous	T7420C	I2385	12.37	15.49	3/17/2020
SRR11494486	orf1a	nonsynonymous	A4291T	A1342S	7.08	3.10	3/17/2020
SRR11494486	orf1a	nonsynonymous	G4289T	A1342S	7.08	3.13	3/17/2020
SRR11494486	orf1a	nonsynonymous	A2826C	N854A	22.64	15.57	3/17/2020
SRR11494486	orf1a	nonsynonymous	A2825G	N854A	22.64	15.57	3/17/2020
SRR11494486	orf1a	nonsynonymous	T2827A	N854A	22.64	16.26	3/17/2020
SRR11494486	orf1a	nonsynonymous	G4285A	E1340K	11.72	8.33	3/17/2020
SRR11494486	orf1a	nonsynonymous	G4283A	E1340K	11.72	9.27	3/17/2020
SRR11494486	orf1a	nonsynonymous	A4293T	K1343M	6.25	3.08	3/17/2020
SRR11494486	orf1a	nonsynonymous	G4286A	E1341I	10.33	8.18	3/17/2020
SRR11494486	orf1a	nonsynonymous	A4287T	E1341I	10.33	6.36	3/17/2020
SRR11494486	orf1a	nonsynonymous	G4288C	E1341I	10.33	6.06	3/17/2020
SRR11494486	orf1a	nonsynonymous	A7626G	N2454S	7.99	6.37	3/17/2020
SRR11494486	orf1a	nonsynonymous	G2835T	C857F	13.79	6.70	3/17/2020
SRR11494486	orf1a	nonsynonymous	T7623G	V2453G	7.57	2.01	3/17/2020
SRR11621999	orf1a	nonsynonymous	T13382G	C4373D	17.00	9.09	04/04/2020
SRR11621999	orf1a	nonsynonymous	G13383A	C4373D	17.00	9.85	04/04/2020
SRR11621999	orf1a	nonsynonymous	G2833C	K856H	6.88	6.37	04/04/2020
SRR11621999	orf1a	nonsynonymous	A2831C	K856H	6.88	8.12	04/04/2020
SRR11621999	orf1a	nonsynonymous	T7623G	V2453G	18.47	4.83	04/04/2020
SRR11621999	orf1a	nonsynonymous	T7436A	Y2391N	8.12	2.21	04/04/2020
SRR11621999	orf1a	nonsynonymous	T2827A	N854A	6.56	3.92	04/04/2020
SRR11621999	orf1a	nonsynonymous	A2825G	N854A	6.56	4.20	04/04/2020
SRR11621999	orf1a	nonsynonymous	A2826C	N854A	6.56	3.79	04/04/2020
SRR11621999	orf1a	nonsynonymous	T2824G	L853Stop	7.03	7.62	04/04/2020
SRR11621999	orf1a	nonsynonymous	C2822T	L853Stop	7.03	4.01	04/04/2020
SRR11621999	orf1a	nonsynonymous	T2823A	L853Stop	7.03	4.16	04/04/2020
SRR11621999	orf1a	nonsynonymous	C13381A	V4372A	12.50	8.39	04/04/2020
SRR11621999	orf1a	nonsynonymous	T13380C	V4372A	12.50	8.05	04/04/2020
SRR11494569	orf1a	nonsynonymous	C4421T	R1386C	10.09	9.08	3/14/2020
SRR11494492	orf1a	nonsynonymous	A1229C	K322Q	8.11	7.85	3/18/2020
SRR11578166	orf1a	nonsynonymous	C635T	R124C	5.91	4.69	3/24/2020
SRR11494578	orf1a	nonsynonymous	A6875T	K2204Y	5.11	3.12	3/14/2020
SRR11494578	orf1a	nonsynonymous	G6877T	K2204Y	5.11	10.29	3/14/2020
SRR11494578	orf1a	nonsynonymous	A7626G	N2454S	6.67	5.42	3/14/2020
SRR11494578	orf1a	nonsynonymous	C2822T	L853Stop	18.82	12.15	3/14/2020
SRR11494578	orf1a	nonsynonymous	T2823A	L853Stop	18.82	12.02	3/14/2020

SRR11494578	orf1a	nonsynonymous	T2824G	L853Stop	18.82	20.38	3/14/2020
SRR11494578	orf1a	nonsynonymous	A2826C	N854A	17.65	11.79	3/14/2020
SRR11494578	orf1a	nonsynonymous	A2825G	N854A	17.65	13.27	3/14/2020
SRR11494578	orf1a	nonsynonymous	T2827A	N854A	17.65	11.68	3/14/2020
SRR11494578	orf1a	synonymous	T2218C	F651	6.14	5.49	3/14/2020
SRR11494656	orf1a	nonsynonymous	G558T	G98V	6.21	5.91	3/23/2020
SRR11494656	orf1a	synonymous	T2839A	S858	5.78	4.55	3/23/2020
SRR11494656	orf1a	nonsynonymous	G9141T	G2959V	5.13	5.39	3/23/2020
SRR11494656	orf1a	nonsynonymous	C6666T	P2134L	5.35	4.63	3/23/2020
SRR11494656	orf1a	nonsynonymous	G7017T	G2251V	6.01	5.83	3/23/2020
SRR11494656	orf1a	nonsynonymous	G2835T	C857F	17.35	9.52	3/23/2020
SRR11494656	orf1a	nonsynonymous	G3564T	G1100V	13.86	12.43	3/23/2020
SRR11494656	orf1a	nonsynonymous	G7038T	G2258V	6.46	5.64	3/23/2020
SRR11494456	orf1a	synonymous	G12331A	R4022	5.48	5.28	3/20/2020
SRR11494456	orf1a	synonymous	T8782C	S2839	20.27	21.95	3/20/2020
SRR11494456	orf1a	nonsynonymous	G13415T	D4384Y	5.49	4.90	3/20/2020
SRR11494644	orf1a	nonsynonymous	T5744C	Y1827H	6.49	4.91	3/23/2020
SRR11494644	orf1a	nonsynonymous	T7941C	V2559A	17.80	21.77	3/23/2020
SRR11494644	orf1a	nonsynonymous	A1345C	L360F	5.63	11.58	3/23/2020
SRR11494644	orf1a	synonymous	T2839A	S858	5.56	3.20	3/23/2020
SRR11494644	orf1a	synonymous	T3526A	S1087	18.18	27.69	3/23/2020
SRR11494644	orf1a	nonsynonymous	C11871G	S3869C	24.81	11.69	3/23/2020
SRR11494644	orf1a	nonsynonymous	A11872T	S3869C	24.81	11.48	3/23/2020
SRR11494644	orf1a	nonsynonymous	T7943G	Y2560D	18.33	24.19	3/23/2020
SRR11494644	orf1a	nonsynonymous	G2835T	C857F	16.28	10.00	3/23/2020
SRR11494644	orf1a	nonsynonymous	T11874C	V3870A	13.60	8.79	3/23/2020
SRR11622071	orf1a	nonsynonymous	T6771C	M2169T	17.21	16.97	04/08/2020
SRR11622071	orf1a	nonsynonymous	T11711C	S3816P	5.43	5.82	04/08/2020
SRR11622071	orf1a	synonymous	C11300T	L3679	32.02	32.27	04/08/2020
SRR11622071	orf1a	synonymous	C8476T	N2737	18.76	19.56	04/08/2020
SRR11622071	orf1a	nonsynonymous	A6863C	K2200Q	11.14	5.06	04/08/2020
SRR11622071	orf1a	nonsynonymous	T2823A	L853Stop	19.70	16.67	04/08/2020
SRR11622071	orf1a	nonsynonymous	C2822T	L853Stop	19.70	16.97	04/08/2020
SRR11622071	orf1a	nonsynonymous	T2824G	L853Stop	19.70	24.13	04/08/2020
SRR11622071	orf1a	nonsynonymous	G1533C	W423S	14.76	14.95	04/08/2020
SRR11622071	orf1a	nonsynonymous	A8116T	E2617D	5.40	4.51	04/08/2020
SRR11622071	orf1a	nonsynonymous	G5384T	A1707S	16.31	15.92	04/08/2020
SRR11622071	orf1a	synonymous	A12307G	K4014	23.76	15.53	04/08/2020
SRR11622071	orf1a	synonymous	T8461C	A2732	17.74	20.75	04/08/2020
SRR11622071	orf1a	nonsynonymous	T8391C	I2709T	20.83	22.12	04/08/2020
SRR11622071	orf1a	nonsynonymous	G3753T	R1163I	7.74	10.00	04/08/2020
SRR11622071	orf1a	nonsynonymous	G13386T	G4374F	16.07	25.20	04/08/2020
SRR11622071	orf1a	nonsynonymous	G13385T	G4374F	16.07	33.86	04/08/2020
SRR11622071	orf1a	synonymous	C13105T	Y4280	23.84	24.10	04/08/2020
SRR11622071	orf1a	nonsynonymous	G4283T	E1340Stop	20.53	24.18	04/08/2020
SRR11622071	orf1a	nonsynonymous	A2825G	N854A	19.38	16.18	04/08/2020

SRR11622071	orf1a	nonsynonymous	T2827A	N854A	19.38	16.76	04/08/2020
SRR11622071	orf1a	nonsynonymous	A2826C	N854A	19.38	15.43	04/08/2020
SRR11622071	orf1a	nonsynonymous	C13381A	V4372A	30.65	8.19	04/08/2020
SRR11622071	orf1a	nonsynonymous	T13380C	V4372A	30.65	7.25	04/08/2020
SRR11622071	orf1a	nonsynonymous	C13005T	A4247V	12.93	2.66	04/08/2020
SRR11622071	orf1a	nonsynonymous	T13006A	A4247V	12.93	7.11	04/08/2020
SRR11622071	orf1a	synonymous	A4282G	V1339	20.68	24.47	04/08/2020
SRR11622071	orf1a	nonsynonymous	A2661G	Y799C	17.04	20.73	04/08/2020
SRR11494457	orf1a	synonymous	C9857T	L3198	14.56	14.17	3/20/2020
SRR11494590	orf1a	synonymous	T2839A	S858	5.26	4.85	3/13/2020
SRR11494590	orf1a	nonsynonymous	A6869T	T2202S	12.33	32.93	3/13/2020
SRR11494590	orf1a	nonsynonymous	A2825G	N854A	27.12	17.57	3/13/2020
SRR11494590	orf1a	nonsynonymous	T2827A	N854A	27.12	18.92	3/13/2020
SRR11494590	orf1a	nonsynonymous	A2826C	N854A	27.12	17.57	3/13/2020
SRR11494590	orf1a	nonsynonymous	A6863C	K2200Q	7.24	3.95	3/13/2020
SRR11494590	orf1a	nonsynonymous	A5283G	N1673S	5.62	5.08	3/13/2020
SRR11494590	orf1a	nonsynonymous	C5284T	N1673S	5.62	4.89	3/13/2020
SRR11494590	orf1a	nonsynonymous	G2835T	C857F	17.27	9.43	3/13/2020
SRR11494590	orf1a	nonsynonymous	G12067A	M3934I	13.01	12.29	3/13/2020
SRR11494590	orf1a	synonymous	T13078C	A4271	16.03	16.41	3/13/2020
SRR11494590	orf1a	nonsynonymous	G2833C	K856H	27.94	18.66	3/13/2020
SRR11494590	orf1a	nonsynonymous	A2831C	K856H	27.94	31.17	3/13/2020
SRR11494576	orf1a	nonsynonymous	C4400T	L1379F	5.65	5.37	3/14/2020
SRR11578349	orf1a	synonymous	G2398A	T711	19.67	19.33	3/29/2020
SRR11494518	orf1a	nonsynonymous	G2835T	C857F	25.37	12.50	3/17/2020
SRR11494518	orf1a	synonymous	T2839A	S858	6.25	3.14	3/17/2020
SRR11494521	orf1a	nonsynonymous	G6513T	S2083I	5.96	4.87	3/17/2020
SRR11494608	orf1a	synonymous	T9223C	H2986	5.37	4.15	3/18/2020
SRR11494608	orf1a	nonsynonymous	T9476A	F3071T	15.28	14.37	3/18/2020
SRR11494608	orf1a	nonsynonymous	T9477C	F3071T	15.28	13.77	3/18/2020
SRR11494468	orf1a	nonsynonymous	T6605C	S2114P	7.05	7.65	3/19/2020
SRR11494618	orf1a	nonsynonymous	G3644A	D1127N	8.29	7.99	03/10/2020
SRR11494616	orf1a	nonsynonymous	A2826C	N854A	17.57	16.85	03/11/2020
SRR11494616	orf1a	nonsynonymous	T2827A	N854A	17.57	16.76	03/11/2020
SRR11494616	orf1a	nonsynonymous	A2825G	N854A	17.57	17.42	03/11/2020
SRR11494616	orf1a	nonsynonymous	T7619G	C2452A	13.01	1.29	03/11/2020
SRR11494616	orf1a	nonsynonymous	G7620C	C2452A	13.01	8.04	03/11/2020
SRR11494616	orf1a	nonsynonymous	C2822T	L853Stop	19.73	17.24	03/11/2020
SRR11494616	orf1a	nonsynonymous	T2823A	L853Stop	19.73	16.95	03/11/2020
SRR11494616	orf1a	nonsynonymous	T2824G	L853Stop	19.73	22.47	03/11/2020
SRR11494616	orf1a	nonsynonymous	T9476C	F3071L	5.26	4.87	03/11/2020
SRR11494616	orf1a	nonsynonymous	G2835T	C857F	11.97	6.58	03/11/2020
SRR11494616	orf1a	nonsynonymous	A6875T	K2204Y	6.10	5.65	03/11/2020
SRR11494616	orf1a	nonsynonymous	G6877T	K2204Y	6.10	12.95	03/11/2020
SRR11494616	orf1a	nonsynonymous	G3995A	E1244K	8.49	7.41	03/11/2020
SRR11494616	orf1a	nonsynonymous	A7626G	N2454S	14.71	15.91	03/11/2020

SRR11494616	orf1a	nonsynonymous	T12804G	F4180C	5.32	4.93	03/11/2020
SRR11494616	orf1a	synonymous	T3412A	A1049	9.88	11.29	03/11/2020
SRR11494616	orf1a	nonsynonymous	T7623G	V2453G	15.47	3.72	03/11/2020
SRR11494648	orf1a	synonymous	A3904T	P1213	12.82	12.10	3/23/2020
SRR11494648	orf1a	nonsynonymous	G11083T	L3606F	7.14	7.72	3/23/2020
SRR11494524	orf1a	synonymous	C11833T	A3856	42.25	42.37	3/17/2020
SRR11494524	orf1a	synonymous	T683C	L140	12.25	11.67	3/17/2020
SRR11494628	orf1a	synonymous	A5317G	Q1684	10.00	10.29	3/24/2020
SRR11494668	orf1a	synonymous	C10369T	R3368	5.59	4.71	3/22/2020
SRR11494653	orf1a	synonymous	C920T	L219	10.15	11.39	3/23/2020
SRR11494653	orf1a	nonsynonymous	G11083T	L3606F	19.48	24.77	3/23/2020
SRR11494454	orf1a	nonsynonymous	G2835T	C857F	27.78	12.12	3/17/2020
SRR11494454	orf1a	synonymous	T2839A	S858	10.29	3.29	3/17/2020
SRR11494619	orf1a	nonsynonymous	T9476A	F3071T	22.36	23.32	3/19/2020
SRR11494619	orf1a	nonsynonymous	T9477C	F3071T	22.36	21.24	3/19/2020
SRR11494619	orf1a	synonymous	C1912T	S549	8.53	7.93	3/19/2020
SRR11578164	orf1a	nonsynonymous	G1406T	E381Stop	5.32	5.18	3/25/2020
SRR11578164	orf1a	nonsynonymous	G11392T	W3709C	6.50	7.33	3/25/2020
SRR11578164	orf1a	nonsynonymous	G10986T	R3574I	13.29	13.17	3/25/2020
SRR11578164	orf1a	nonsynonymous	G7246T	W2327C	14.56	14.31	3/25/2020
SRR11578164	orf1a	synonymous	G13240T	S4325	6.83	6.77	3/25/2020
SRR11578164	orf1a	nonsynonymous	C13239T	S4325L	5.39	4.99	3/25/2020
SRR11578164	orf1a	nonsynonymous	G7017T	G2251V	5.65	5.33	3/25/2020
SRR11578164	orf1a	nonsynonymous	G11535T	G3757V	13.33	12.71	3/25/2020
SRR11578164	orf1a	nonsynonymous	C6468T	T2068I	5.33	6.54	3/25/2020
SRR11578164	orf1a	nonsynonymous	C3551T	P1096S	8.40	7.97	3/25/2020
SRR11578164	orf1a	nonsynonymous	G6804T	C2180F	5.28	5.25	3/25/2020
SRR11578164	orf1a	nonsynonymous	G7363T	W2366C	7.03	6.80	3/25/2020
SRR11578164	orf1a	nonsynonymous	G8790T	G2842V	16.22	15.85	3/25/2020
SRR11578164	orf1a	nonsynonymous	G2036T	A591S	10.28	9.89	3/25/2020
SRR11578164	orf1a	nonsynonymous	G10754T	A3497S	6.94	5.46	3/25/2020
SRR11578164	orf1a	nonsynonymous	G3004T	E913D	14.20	14.20	3/25/2020
SRR11578164	orf1a	nonsynonymous	C1968A	T568Stop	5.07	6.76	3/25/2020
SRR11578164	orf1a	nonsynonymous	A1967T	T568Stop	5.07	4.90	3/25/2020
SRR11578164	orf1a	nonsynonymous	G9141T	G2959V	16.10	16.96	3/25/2020
SRR11578164	orf1a	nonsynonymous	G5375T	A1704S	13.44	13.26	3/25/2020
SRR11578164	orf1a	nonsynonymous	C482T	R73C	8.48	9.34	3/25/2020
SRR11578164	orf1a	nonsynonymous	G8696T	A2811S	5.01	4.78	3/25/2020
SRR11578164	orf1a	nonsynonymous	G8688T	G2808V	10.38	14.28	3/25/2020
SRR11578164	orf1a	nonsynonymous	G4505T	G1414C	11.18	10.96	3/25/2020
SRR11578164	orf1a	synonymous	C4832T	L1523	7.17	7.19	3/25/2020
SRR11578164	orf1a	nonsynonymous	G7214T	D2317Y	6.26	6.05	3/25/2020
SRR11578164	orf1a	nonsynonymous	C12141T	T3959I	6.26	6.82	3/25/2020
SRR11578164	orf1a	nonsynonymous	G7038T	G2258V	25.55	26.16	3/25/2020
SRR11578164	orf1a	nonsynonymous	G5378T	G1705C	6.07	6.33	3/25/2020
SRR11578164	orf1a	nonsynonymous	G13193T	V4310F	7.23	7.33	3/25/2020

SRR11494537	orf1a	nonsynonymous	A7626G	N2454S	9.50	7.05	02/02/2020
SRR11494537	orf1a	nonsynonymous	G10862A	E3533K	6.29	5.46	02/02/2020
SRR11494537	orf1a	nonsynonymous	A6875T	K2204Y	8.43	3.14	02/02/2020
SRR11494537	orf1a	nonsynonymous	G6877T	K2204Y	8.43	11.44	02/02/2020
SRR11494537	orf1a	nonsynonymous	C7269T	T2335I	5.07	4.42	02/02/2020
SRR11494607	orf1a	nonsynonymous	C401T	L46F	19.62	21.70	03/11/2020
SRR11494683	orf1b	nonsynonymous	C16289T	A941V	21.99	22.44	3/22/2020
SRR11494762	orf1b	synonymous	T15462C	Y665	5.82	5.87	2/21/2020
SRR11494762	orf1b	nonsynonymous	G20680T	G2405C	6.08	5.52	2/21/2020
SRR11622072	orf1b	nonsynonymous	T16835A	F1123Y	7.94	0.48	04/07/2020
SRR11622072	orf1b	nonsynonymous	G14974T	A503S	34.09	33.99	04/07/2020
SRR11622072	orf1b	synonymous	A16824T	G1119	5.85	2.69	04/07/2020
SRR11622072	orf1b	nonsynonymous	G20477T	G2337V	15.15	13.87	04/07/2020
SRR11622072	orf1b	nonsynonymous	A13780T	I105L	15.76	16.08	04/07/2020
SRR11622072	orf1b	nonsynonymous	A16831T	T1122S	8.51	12.25	04/07/2020
SRR11622072	orf1b	nonsynonymous	A21163T	I2566L	7.14	8.28	04/07/2020
SRR11494497	orf1b	synonymous	T14451C	G328	16.48	15.36	3/17/2020
SRR11494657	orf1b	nonsynonymous	G16988T	G1174V	5.06	5.03	3/23/2020
SRR11494657	orf1b	nonsynonymous	G17848T	G1461C	10.45	9.64	3/23/2020
SRR11494657	orf1b	nonsynonymous	C14536T	L357F	6.49	6.58	3/23/2020
SRR11494657	orf1b	nonsynonymous	G16068T	E867D	6.75	7.77	3/23/2020
SRR11494657	orf1b	nonsynonymous	G15769T	A768S	6.27	6.48	3/23/2020
SRR11494657	orf1b	nonsynonymous	G20128T	E2221Stop	6.13	6.00	3/23/2020
SRR11494657	orf1b	nonsynonymous	G13571T	G35V	18.43	16.21	3/23/2020
SRR11494657	orf1b	nonsynonymous	G16508T	G1014V	5.96	5.64	3/23/2020
SRR11494569	orf1b	nonsynonymous	C16733T	S1089L	14.02	14.56	3/14/2020
SRR11494467	orf1b	synonymous	C20719T	L2418	6.32	6.47	03/08/2020
SRR11494460	orf1b	synonymous	C17550T	L1361	18.48	18.41	3/19/2020
SRR11494460	orf1b	nonsynonymous	G17122T	A1219S	13.45	12.36	3/19/2020
SRR11494555	orf1b	nonsynonymous	A21550C	N2695L	8.46	6.29	3/16/2020
SRR11494555	orf1b	nonsynonymous	A21551T	N2695L	8.46	7.52	3/16/2020
SRR11494555	orf1b	nonsynonymous	A21548T	N2694V	9.07	6.02	3/16/2020
SRR11494555	orf1b	nonsynonymous	A21547G	N2694V	9.07	6.19	3/16/2020
SRR11494555	orf1b	nonsynonymous	C21549T	N2694V	9.07	6.21	3/16/2020
SRR11494557	orf1b	nonsynonymous	T17500A	F1345I	7.46	2.02	3/15/2020
SRR11494557	orf1b	nonsynonymous	G19947C	K2160N	8.49	6.63	3/15/2020
SRR11494557	orf1b	synonymous	T13668C	T67	6.77	7.98	3/15/2020
SRR11494557	orf1b	nonsynonymous	G19932C	M2155V	8.53	7.28	3/15/2020
SRR11494557	orf1b	nonsynonymous	A19930G	M2155V	8.53	6.38	3/15/2020
SRR11494557	orf1b	nonsynonymous	G19942A	A2159T	6.88	6.45	3/15/2020
SRR11494557	orf1b	nonsynonymous	C19944A	A2159T	6.88	7.69	3/15/2020
SRR11494557	orf1b	nonsynonymous	A21550C	N2695L	10.67	7.65	3/15/2020
SRR11494557	orf1b	nonsynonymous	A21551T	N2695L	10.67	8.18	3/15/2020
SRR11494557	orf1b	nonsynonymous	T19940G	I2158R	7.85	6.54	3/15/2020
SRR11494557	orf1b	synonymous	A17487T	L1340	5.67	0.15	3/15/2020
SRR11494557	orf1b	nonsynonymous	T17752A	W1429T	28.05	3.68	3/15/2020

SRR11494557	orf1b	nonsynonymous	G17753C	W1429T	28.05	10.88	3/15/2020
SRR11494557	orf1b	nonsynonymous	G17754T	W1429T	28.05	11.74	3/15/2020
SRR11494557	orf1b	nonsynonymous	C19934G	T2156S	9.75	7.24	3/15/2020
SRR11494557	orf1b	nonsynonymous	C21549T	N2694V	11.18	6.65	3/15/2020
SRR11494557	orf1b	nonsynonymous	A21547G	N2694V	11.18	6.63	3/15/2020
SRR11494557	orf1b	nonsynonymous	A21548T	N2694V	11.18	7.42	3/15/2020
SRR11494557	orf1b	nonsynonymous	C17740G	R1425G	8.46	0.13	3/15/2020
SRR11494557	orf1b	nonsynonymous	T17742G	R1425G	8.46	0.21	3/15/2020
SRR11494557	orf1b	nonsynonymous	G19936C	D2157H	9.32	6.98	3/15/2020
SRR11494557	orf1b	nonsynonymous	C19938T	D2157H	9.32	6.54	3/15/2020
SRR11621993	orf1b	nonsynonymous	A15248G	K594R	14.58	14.84	04/01/2020
SRR11621993	orf1b	synonymous	G18021A	R1518	5.72	5.46	04/01/2020
SRR11621993	orf1b	synonymous	A13689G	E74	5.02	5.53	04/01/2020
SRR11621993	orf1b	nonsynonymous	G19947C	K2160N	9.59	9.30	04/01/2020
SRR11621993	orf1b	nonsynonymous	T16828C	Y1121L	7.81	6.25	04/01/2020
SRR11621993	orf1b	nonsynonymous	A16829T	Y1121L	7.81	6.64	04/01/2020
SRR11621993	orf1b	nonsynonymous	G19942A	A2159T	9.44	8.92	04/01/2020
SRR11621993	orf1b	nonsynonymous	C19944A	A2159T	9.44	10.30	04/01/2020
SRR11621993	orf1b	nonsynonymous	A16831T	T1122S	7.81	10.01	04/01/2020
SRR11621993	orf1b	nonsynonymous	C19938T	D2157H	10.19	8.44	04/01/2020
SRR11621993	orf1b	nonsynonymous	G19936C	D2157H	10.19	8.53	04/01/2020
SRR11621993	orf1b	nonsynonymous	G18007A	E1514K	5.13	3.53	04/01/2020
SRR11621993	orf1b	synonymous	A16824T	G1119	5.33	1.77	04/01/2020
SRR11621993	orf1b	nonsynonymous	T19940G	I2158R	9.33	9.09	04/01/2020
SRR11494607	orf1b	nonsynonymous	C16733T	S1089L	19.92	21.01	03/11/2020
SRR11494539	orf1b	synonymous	T14313C	D282	10.43	11.33	2/23/2020
SRR11494708	orf1b	nonsynonymous	A21137G	K2557R	5.35	5.16	3/18/2020
SRR11494626	orf1b	nonsynonymous	G13993T	A176S	7.83	6.80	3/24/2020
SRR11494648	orf1b	synonymous	C17304T	V1279	11.47	11.33	3/23/2020
SRR11494519	orf1b	nonsynonymous	T19940G	I2158R	5.21	2.52	3/16/2020
SRR11494519	orf1b	nonsynonymous	G19932C	M2155V	5.02	2.93	3/16/2020
SRR11494519	orf1b	nonsynonymous	A19930G	M2155V	5.02	2.57	3/16/2020
SRR11494519	orf1b	nonsynonymous	A21548T	N2694V	6.24	4.01	3/16/2020
SRR11494519	orf1b	nonsynonymous	A21547G	N2694V	6.24	3.54	3/16/2020
SRR11494519	orf1b	nonsynonymous	C21549T	N2694V	6.24	3.54	3/16/2020
SRR11494519	orf1b	nonsynonymous	G19947C	K2160N	5.02	2.41	3/16/2020
SRR11494519	orf1b	nonsynonymous	G19936C	D2157H	5.25	2.55	3/16/2020
SRR11494519	orf1b	nonsynonymous	C19938T	D2157H	5.25	2.52	3/16/2020
SRR11494565	orf1b	synonymous	G17562T	R1365	9.83	9.62	3/15/2020
SRR11494663	orf1b	synonymous	C17373T	A1302	32.52	32.21	3/19/2020
SRR11494653	orf1b	synonymous	T14466C	V333	7.08	6.64	3/23/2020
SRR11621999	orf1b	nonsynonymous	G16827A	E1120V	8.33	6.72	04/04/2020
SRR11621999	orf1b	nonsynonymous	A16826T	E1120V	8.33	8.91	04/04/2020
SRR11621999	orf1b	nonsynonymous	T16828C	Y1121L	8.92	6.79	04/04/2020
SRR11621999	orf1b	nonsynonymous	A16829T	Y1121L	8.92	6.99	04/04/2020
SRR11621999	orf1b	nonsynonymous	G13571T	G35V	8.33	6.84	04/04/2020

SRR11621999	orf1b	nonsynonymous	A16831T	T1122S	8.95	9.03	04/04/2020
SRR11621999	orf1b	nonsynonymous	A21166T	K2567Stop	12.86	16.85	04/04/2020
SRR11621999	orf1b	synonymous	A16824T	G1119	5.72	2.04	04/04/2020
SRR11494603	orf1b	synonymous	T18060C	L1531	16.54	15.34	03/12/2020
SRR11494615	orf1b	nonsynonymous	G20887A	G2474R	45.23	45.81	03/11/2020
SRR11622002	orf1b	nonsynonymous	A21166T	K2567Stop	7.69	14.07	04/03/2020
SRR11622002	orf1b	synonymous	T13638A	I57	36.54	41.40	04/03/2020
SRR11494494	orf1b	synonymous	C17586T	D1373	10.59	10.84	3/18/2020
SRR11494494	orf1b	nonsynonymous	A19175G	D1903G	25.87	25.35	3/18/2020
SRR11578317	orf1b	nonsynonymous	C18131T	T1555I	6.77	5.98	3/27/2020
SRR11494594	orf1b	nonsynonymous	G18997A	A1844T	6.91	6.70	03/12/2020
SRR11578167	orf1b	nonsynonymous	G19936C	D2157H	5.63	3.06	3/24/2020
SRR11578167	orf1b	nonsynonymous	C19938T	D2157H	5.63	2.97	3/24/2020
SRR11578167	orf1b	nonsynonymous	T19940G	I2158R	5.05	2.97	3/24/2020
SRR11578167	orf1b	nonsynonymous	G19932C	M2155V	5.26	2.93	3/24/2020
SRR11578167	orf1b	nonsynonymous	A19930G	M2155V	5.26	2.91	3/24/2020
SRR11578167	orf1b	nonsynonymous	C19944A	A2159T	5.18	3.46	3/24/2020
SRR11578167	orf1b	nonsynonymous	G19942A	A2159T	5.18	2.84	3/24/2020
SRR11578167	orf1b	nonsynonymous	C19934G	T2156S	5.59	3.01	3/24/2020
SRR11494538	orf1b	nonsynonymous	C17678T	T1404M	12.57	13.08	3/15/2020
SRR11494523	orf1b	synonymous	C18060T	L1531	19.30	16.94	3/17/2020
SRR11494523	orf1b	nonsynonymous	A19013G	K1849R	11.25	10.00	3/17/2020
SRR11494523	orf1b	nonsynonymous	C17747T	P1427L	10.00	5.65	3/17/2020
SRR11494523	orf1b	synonymous	T14805C	Y446	12.56	11.07	3/17/2020
SRR11494523	orf1b	nonsynonymous	G20709T	M2414I	5.81	6.06	3/17/2020
SRR11494523	orf1b	synonymous	C17247T	R1260	6.26	6.83	3/17/2020
SRR11494459	orf1b	synonymous	C19017T	F1850	5.33	5.70	3/19/2020
SRR11494556	orf1b	nonsynonymous	T17747C	P1427P	15.29	25.40	3/16/2020
SRR11494652	orf1b	synonymous	C17550T	L1361	21.82	21.96	3/19/2020
SRR11494679	orf1b	synonymous	T15102A	A545	6.99	6.63	03/10/2020
SRR11494620	orf1b	nonsynonymous	G19947C	K2160N	16.44	13.68	03/10/2020
SRR11494620	orf1b	nonsynonymous	C19938T	D2157H	18.10	10.60	03/10/2020
SRR11494620	orf1b	nonsynonymous	G19936C	D2157H	18.10	11.39	03/10/2020
SRR11494620	orf1b	nonsynonymous	C19944A	A2159T	17.27	16.30	03/10/2020
SRR11494620	orf1b	nonsynonymous	G19942A	A2159T	17.27	10.99	03/10/2020
SRR11494620	orf1b	nonsynonymous	A19930G	M2155V	15.77	9.97	03/10/2020
SRR11494620	orf1b	nonsynonymous	G19932C	M2155V	15.77	9.84	03/10/2020
SRR11494620	orf1b	nonsynonymous	C19934G	T2156S	17.31	10.58	03/10/2020
SRR11494620	orf1b	nonsynonymous	T19940G	I2158R	16.97	10.95	03/10/2020
SRR11494761	orf1b	nonsynonymous	C19868T	T2134I	5.60	4.72	3/19/2020
SRR11494650	orf1b	synonymous	G17100A	K1211	16.50	20.00	3/23/2020
SRR11494590	orf1b	synonymous	A15702T	S745	9.89	10.12	3/13/2020
SRR11494590	orf1b	nonsynonymous	T19420C	S1985P	12.05	13.02	3/13/2020
SRR11494486	orf1b	nonsynonymous	G19936C	D2157H	6.16	4.02	3/17/2020
SRR11494486	orf1b	nonsynonymous	C19938T	D2157H	6.16	3.99	3/17/2020
SRR11494486	orf1b	nonsynonymous	C13495A	L10I	17.07	11.36	3/17/2020

SRR11494486	orf1b	nonsynonymous	A20296G	K2277V	6.28	6.81	3/17/2020
SRR11494486	orf1b	nonsynonymous	A20297T	K2277V	6.28	7.14	3/17/2020
SRR11494486	orf1b	nonsynonymous	G19947C	K2160N	5.50	4.03	3/17/2020
SRR11494486	orf1b	nonsynonymous	A19930G	M2155V	5.64	3.92	3/17/2020
SRR11494486	orf1b	nonsynonymous	G19932C	M2155V	5.64	3.95	3/17/2020
SRR11494486	orf1b	nonsynonymous	C19934G	T2156S	5.98	4.05	3/17/2020
SRR11494486	orf1b	nonsynonymous	T19940G	I2158R	5.05	3.98	3/17/2020
SRR11494486	orf1b	nonsynonymous	T20300A	L2278Stop	6.80	6.93	3/17/2020
SRR11621987	orf1b	nonsynonymous	A16460G	H998R	5.84	6.78	04/01/2020
SRR11621987	orf1b	nonsynonymous	C19944A	A2159T	13.18	14.29	04/01/2020
SRR11621987	orf1b	nonsynonymous	G19942A	A2159T	13.18	10.13	04/01/2020
SRR11621987	orf1b	nonsynonymous	A16829T	Y1121L	10.67	9.55	04/01/2020
SRR11621987	orf1b	nonsynonymous	T16828C	Y1121L	10.67	9.09	04/01/2020
SRR11621987	orf1b	nonsynonymous	T17510G	V1348G	8.96	9.09	04/01/2020
SRR11621987	orf1b	nonsynonymous	T19940G	I2158R	13.64	10.26	04/01/2020
SRR11621987	orf1b	synonymous	C14790T	I441	7.35	6.65	04/01/2020
SRR11621987	orf1b	nonsynonymous	A16831T	T1122S	10.69	12.89	04/01/2020
SRR11621987	orf1b	nonsynonymous	G19947C	K2160N	14.18	9.82	04/01/2020
SRR11621987	orf1b	nonsynonymous	A18982T	M1839L	7.66	0.55	04/01/2020
SRR11621987	orf1b	nonsynonymous	C19934G	T2156S	13.39	9.64	04/01/2020
SRR11621987	orf1b	synonymous	A16824T	G1119	7.05	2.07	04/01/2020
SRR11494559	orf1b	synonymous	T17247C	R1260	5.32	5.93	3/15/2020
SRR11494559	orf1b	synonymous	C14805T	Y446	6.53	6.81	3/15/2020
SRR11494559	orf1b	nonsynonymous	T14408C	P314P	7.17	7.37	3/15/2020
SRR11494559	orf1b	synonymous	G20268A	L2267	8.66	7.13	3/15/2020
SRR11494559	orf1b	synonymous	A17799G	V1444	6.42	6.42	3/15/2020
SRR11494515	orf1b	synonymous	T14877C	Y470	9.80	8.89	3/18/2020
SRR11494623	orf1b	synonymous	T14805C	Y446	25.17	23.49	3/24/2020
SRR11494623	orf1b	nonsynonymous	T13627G	D54D	27.41	27.80	3/24/2020
SRR11494654	orf1b	nonsynonymous	T19788G	E2107E	34.27	35.51	03/10/2020
SRR11494578	orf1b	nonsynonymous	A13635T	L56F	10.68	13.60	3/14/2020
SRR11494578	orf1b	nonsynonymous	C19938T	D2157H	5.61	3.62	3/14/2020
SRR11494578	orf1b	nonsynonymous	G19936C	D2157H	5.61	3.47	3/14/2020
SRR11494578	orf1b	nonsynonymous	A13636T	I57F	10.05	13.56	3/14/2020
SRR11494645	orf1b	nonsynonymous	G13571T	G35V	14.75	13.72	3/23/2020
SRR11494645	orf1b	nonsynonymous	G17848T	G1461C	5.32	5.56	3/23/2020
SRR11494645	orf1b	nonsynonymous	G16508T	G1014V	5.79	6.57	3/23/2020
SRR11494645	orf1b	nonsynonymous	C14536T	L357F	5.12	5.00	3/23/2020
SRR11494645	orf1b	nonsynonymous	G15769T	A768S	8.16	8.25	3/23/2020
SRR11494577	orf1b	nonsynonymous	G19947C	K2160N	5.00	3.57	3/14/2020
SRR11494577	orf1b	nonsynonymous	A19930G	M2155V	5.09	3.57	3/14/2020
SRR11494577	orf1b	nonsynonymous	G19932C	M2155V	5.09	3.47	3/14/2020
SRR11494577	orf1b	nonsynonymous	C21549T	N2694V	7.97	5.48	3/14/2020
SRR11494577	orf1b	nonsynonymous	A21548T	N2694V	7.97	5.26	3/14/2020
SRR11494577	orf1b	nonsynonymous	A21547G	N2694V	7.97	5.64	3/14/2020
SRR11494577	orf1b	nonsynonymous	C19938T	D2157H	5.15	3.79	3/14/2020

SRR11494577	orf1b	nonsynonymous	G19936C	D2157H	5.15	3.81	3/14/2020
SRR11494577	orf1b	nonsynonymous	A21551T	N2695L	8.43	6.62	3/14/2020
SRR11494577	orf1b	nonsynonymous	A21550C	N2695L	8.43	5.69	3/14/2020
SRR11578323	orf1b	nonsynonymous	C19944A	A2159T	7.35	7.11	3/28/2020
SRR11578323	orf1b	nonsynonymous	G19942A	A2159T	7.35	4.70	3/28/2020
SRR11578323	orf1b	nonsynonymous	A19930G	M2155V	6.71	4.64	3/28/2020
SRR11578323	orf1b	nonsynonymous	G19932C	M2155V	6.71	4.87	3/28/2020
SRR11578323	orf1b	nonsynonymous	A16829T	Y1121L	6.16	4.05	3/28/2020
SRR11578323	orf1b	nonsynonymous	T16828C	Y1121L	6.16	3.78	3/28/2020
SRR11578323	orf1b	nonsynonymous	C19934G	T2156S	8.35	4.97	3/28/2020
SRR11578323	orf1b	nonsynonymous	A16831T	T1122S	5.87	5.17	3/28/2020
SRR11578323	orf1b	nonsynonymous	T19940G	I2158R	6.96	4.75	3/28/2020
SRR11578323	orf1b	nonsynonymous	G19947C	K2160N	6.57	4.95	3/28/2020
SRR11578323	orf1b	nonsynonymous	G16827A	E1120V	5.80	3.79	3/28/2020
SRR11578323	orf1b	nonsynonymous	A16826T	E1120V	5.80	5.21	3/28/2020
SRR11578323	orf1b	nonsynonymous	G19936C	D2157H	8.35	4.95	3/28/2020
SRR11578323	orf1b	nonsynonymous	C19938T	D2157H	8.35	4.92	3/28/2020
SRR11578323	orf1b	nonsynonymous	T13648C	F61L	8.70	8.06	3/28/2020
SRR11494548	orf1b	nonsynonymous	A21551T	N2695L	5.69	4.11	1/31/2020
SRR11494548	orf1b	nonsynonymous	A21550C	N2695L	5.69	3.18	1/31/2020
SRR11494591	orf1b	synonymous	A18180G	K1571	10.88	12.48	3/13/2020
SRR11494491	orf1b	synonymous	C13585T	L40	6.41	5.62	3/18/2020
SRR11494613	orf1b	synonymous	A14004G	K179	5.15	5.72	03/11/2020
SRR11494514	orf1b	nonsynonymous	G20773T	G2436C	12.05	10.92	3/18/2020
SRR11494597	orf1b	nonsynonymous	C20629T	H2388Y	17.59	17.25	3/20/2020
SRR11622071	orf1b	synonymous	C21108T	F2547	8.56	2.26	04/08/2020
SRR11622071	orf1b	nonsynonymous	A15239T	N591M	9.09	17.65	04/08/2020
SRR11622071	orf1b	nonsynonymous	C15240G	N591M	9.09	9.23	04/08/2020
SRR11622071	orf1b	nonsynonymous	G20933T	G2489V	48.08	49.18	04/08/2020
SRR11622071	orf1b	nonsynonymous	C20978T	A2504V	23.02	24.84	04/08/2020
SRR11622071	orf1b	nonsynonymous	T17003A	L1179H	14.21	14.26	04/08/2020
SRR11622071	orf1b	nonsynonymous	T19940G	I2158R	8.58	7.67	04/08/2020
SRR11622071	orf1b	nonsynonymous	A15241T	M592L	16.22	17.02	04/08/2020
SRR11622071	orf1b	nonsynonymous	G19936C	D2157H	10.55	7.69	04/08/2020
SRR11622071	orf1b	nonsynonymous	C19938T	D2157H	10.55	7.95	04/08/2020
SRR11622071	orf1b	synonymous	A19368T	P1967	11.48	10.30	04/08/2020
SRR11622071	orf1b	nonsynonymous	A20662T	S2399C	6.38	5.18	04/08/2020
SRR11622071	orf1b	nonsynonymous	G15487T	G674Stop	5.22	7.44	04/08/2020
SRR11622071	orf1b	synonymous	A15486C	S673	7.59	7.10	04/08/2020
SRR11494456	orf1b	synonymous	C19185T	C1906	25.83	26.15	3/20/2020
SRR11494585	orf1b	nonsynonymous	C19934G	T2156S	11.17	7.49	3/13/2020
SRR11494585	orf1b	nonsynonymous	G19932C	M2155V	10.39	6.76	3/13/2020
SRR11494585	orf1b	nonsynonymous	A19930G	M2155V	10.39	6.44	3/13/2020
SRR11494585	orf1b	nonsynonymous	C19944A	A2159T	11.01	9.54	3/13/2020
SRR11494585	orf1b	nonsynonymous	G19942A	A2159T	11.01	7.14	3/13/2020
SRR11494585	orf1b	nonsynonymous	T19940G	I2158R	10.86	7.25	3/13/2020

SRR11494585	orf1b	nonsynonymous	G19947C	K2160N	11.36	8.58	3/13/2020
SRR11494585	orf1b	nonsynonymous	C19938T	D2157H	12.20	7.25	3/13/2020
SRR11494585	orf1b	nonsynonymous	G19936C	D2157H	12.20	7.39	3/13/2020
SRR11494585	orf1b	nonsynonymous	G14653T	V396F	7.08	7.48	3/13/2020
SRR11494585	orf1b	nonsynonymous	G21354T	R2629S	5.84	5.16	3/13/2020
SRR11494585	orf1b	nonsynonymous	A16890T	K1141N	5.80	6.24	3/13/2020
SRR11578322	orf1b	nonsynonymous	G19936C	D2157H	10.59	5.71	3/28/2020
SRR11578322	orf1b	nonsynonymous	C19938T	D2157H	10.59	5.47	3/28/2020
SRR11578322	orf1b	nonsynonymous	A18008G	E1514G	31.00	27.58	3/28/2020
SRR11578322	orf1b	nonsynonymous	G19947C	K2160N	9.45	6.14	3/28/2020
SRR11578322	orf1b	nonsynonymous	A16831T	T1122S	7.08	6.73	3/28/2020
SRR11578322	orf1b	nonsynonymous	T19940G	I2158R	9.44	5.50	3/28/2020
SRR11578322	orf1b	nonsynonymous	G19942A	A2159T	8.71	5.45	3/28/2020
SRR11578322	orf1b	nonsynonymous	C19944A	A2159T	8.71	9.33	3/28/2020
SRR11578322	orf1b	nonsynonymous	A19930G	M2155V	8.90	5.02	3/28/2020
SRR11578322	orf1b	nonsynonymous	G19932C	M2155V	8.90	5.26	3/28/2020
SRR11578322	orf1b	nonsynonymous	T16828C	Y1121L	6.78	5.19	3/28/2020
SRR11578322	orf1b	nonsynonymous	A16829T	Y1121L	6.78	5.45	3/28/2020
SRR11578322	orf1b	nonsynonymous	C19934G	T2156S	9.65	5.28	3/28/2020
SRR11578318	orf1b	nonsynonymous	C19938T	D2157H	8.91	5.02	3/27/2020
SRR11578318	orf1b	nonsynonymous	G19936C	D2157H	8.91	5.08	3/27/2020
SRR11578318	orf1b	nonsynonymous	T19940G	I2158R	7.72	5.14	3/27/2020
SRR11578318	orf1b	nonsynonymous	C19934G	T2156S	8.41	5.15	3/27/2020
SRR11578318	orf1b	nonsynonymous	G19932C	M2155V	7.61	5.02	3/27/2020
SRR11578318	orf1b	nonsynonymous	A19930G	M2155V	7.61	4.89	3/27/2020
SRR11578318	orf1b	nonsynonymous	G19942A	A2159T	7.18	5.09	3/27/2020
SRR11578318	orf1b	nonsynonymous	C19944A	A2159T	7.18	6.57	3/27/2020
SRR11578318	orf1b	nonsynonymous	G19947C	K2160N	8.06	5.27	3/27/2020
SRR11578318	orf1b	nonsynonymous	C17288T	T1274I	7.00	7.49	3/27/2020
SRR11494513	orf1b	nonsynonymous	A16076G	D870G	11.22	11.60	3/18/2020
SRR11494571	orf1b	synonymous	C14724T	F419	43.23	45.02	3/14/2020
SRR11494592	orf1b	synonymous	C17850T	G1461	5.34	5.63	3/13/2020
SRR11494616	orf1b	nonsynonymous	C19938T	D2157H	12.62	9.68	03/11/2020
SRR11494616	orf1b	nonsynonymous	G19936C	D2157H	12.62	9.68	03/11/2020
SRR11494616	orf1b	nonsynonymous	G19947C	K2160N	12.15	10.04	03/11/2020
SRR11494616	orf1b	nonsynonymous	T19940G	I2158R	11.17	10.08	03/11/2020
SRR11494616	orf1b	nonsynonymous	C19934G	T2156S	11.65	9.47	03/11/2020
SRR11494616	orf1b	nonsynonymous	A19930G	M2155V	11.56	8.80	03/11/2020
SRR11494616	orf1b	nonsynonymous	G19932C	M2155V	11.56	8.89	03/11/2020
SRR11494616	orf1b	nonsynonymous	C19944A	A2159T	10.78	12.40	03/11/2020
SRR11494616	orf1b	nonsynonymous	G19942A	A2159T	10.78	10.04	03/11/2020
SRR11494537	orf1b	nonsynonymous	C19938T	D2157H	7.38	5.75	02/02/2020
SRR11494537	orf1b	nonsynonymous	G19936C	D2157H	7.38	5.75	02/02/2020
SRR11494537	orf1b	nonsynonymous	G19947C	K2160N	6.82	5.63	02/02/2020
SRR11494537	orf1b	nonsynonymous	T19940G	I2158R	6.95	5.71	02/02/2020
SRR11494537	orf1b	nonsynonymous	C19934G	T2156S	7.20	5.51	02/02/2020

SRR11494537	orf1b	nonsynonymous	A18887G	H1807R	5.12	4.87	02/02/2020
SRR11494537	orf1b	nonsynonymous	G19932C	M2155V	6.34	5.19	02/02/2020
SRR11494537	orf1b	nonsynonymous	A19930G	M2155V	6.34	5.00	02/02/2020
SRR11494537	orf1b	nonsynonymous	C19944A	A2159T	5.95	5.99	02/02/2020
SRR11494537	orf1b	nonsynonymous	G19942A	A2159T	5.95	5.99	02/02/2020
SRR11578164	orf1b	nonsynonymous	C14536T	L357F	6.80	9.15	3/25/2020
SRR11578164	orf1b	nonsynonymous	G17668T	G1401C	6.23	5.61	3/25/2020
SRR11578164	orf1b	nonsynonymous	G13571T	G35V	12.69	11.88	3/25/2020
SRR11578164	orf1b	nonsynonymous	G14548A	E361K	6.19	7.74	3/25/2020
SRR11578164	orf1b	nonsynonymous	G17849T	G1461V	7.79	7.42	3/25/2020
SRR11578164	orf1b	nonsynonymous	G17848T	G1461C	5.74	6.49	3/25/2020
SRR11494604	orf1b	nonsynonymous	C21301A	P2612T	48.93	48.29	03/12/2020
SRR11494516	S	nonsynonymous	A21630G	Q23R	30.19	30.28	3/17/2020
SRR11494580	S	nonsynonymous	G23755T	M731I	31.89	32.26	3/13/2020
SRR11494580	S	nonsynonymous	A21650G	N30G	5.40	5.26	3/13/2020
SRR11494580	S	nonsynonymous	A21651G	N30G	5.40	5.28	3/13/2020
SRR11494580	S	nonsynonymous	T21652G	N30G	5.40	5.70	3/13/2020
SRR11494580	S	nonsynonymous	G21665C	G35R	6.02	6.50	3/13/2020
SRR11494580	S	nonsynonymous	G21663C	R34P	6.13	6.07	3/13/2020
SRR11494580	S	nonsynonymous	T21664A	R34P	6.13	6.39	3/13/2020
SRR11494580	S	nonsynonymous	T21657A	F32Stop	5.78	6.62	3/13/2020
SRR11494580	S	nonsynonymous	C21658A	F32Stop	5.78	6.39	3/13/2020
SRR11494580	S	nonsynonymous	T21655A	S31Stop	5.04	6.62	3/13/2020
SRR11494580	S	nonsynonymous	C21654A	S31Stop	5.04	6.39	3/13/2020
SRR11494616	S	synonymous	G22471A	L303	5.26	4.92	03/11/2020
SRR11494616	S	nonsynonymous	G21665C	G35R	5.09	3.95	03/11/2020
SRR11578164	S	nonsynonymous	G23162T	V534F	9.32	8.50	3/25/2020
SRR11578164	S	nonsynonymous	G24557T	G999C	13.50	13.10	3/25/2020
SRR11578164	S	nonsynonymous	C23766T	S735L	6.13	7.31	3/25/2020
SRR11578164	S	nonsynonymous	G22899T	G446V	18.27	15.38	3/25/2020
SRR11578164	S	nonsynonymous	C23855T	R765C	5.81	5.09	3/25/2020
SRR11494519	S	nonsynonymous	T21657A	F32Stop	5.35	6.33	3/16/2020
SRR11494519	S	nonsynonymous	C21658A	F32Stop	5.35	7.13	3/16/2020
SRR11494519	S	nonsynonymous	T21664A	R34P	5.97	6.89	3/16/2020
SRR11494519	S	nonsynonymous	G21663C	R34P	5.97	6.20	3/16/2020
SRR11494519	S	nonsynonymous	G21665C	G35R	6.48	6.91	3/16/2020
SRR11494519	S	nonsynonymous	A21650G	N30G	5.41	5.91	3/16/2020
SRR11494519	S	nonsynonymous	T21652G	N30G	5.41	5.94	3/16/2020
SRR11494519	S	nonsynonymous	A21651G	N30G	5.41	5.95	3/16/2020
SRR11494679	S	nonsynonymous	G21663C	R34P	5.02	5.06	03/10/2020
SRR11494679	S	nonsynonymous	T21664A	R34P	5.02	5.96	03/10/2020
SRR11494679	S	nonsynonymous	G21665C	G35R	5.30	5.39	03/10/2020
SRR11494596	S	nonsynonymous	G21665C	G35R	5.30	5.35	03/12/2020
SRR11494585	S	nonsynonymous	G21665C	G35R	9.76	9.83	3/13/2020
SRR11494585	S	nonsynonymous	A21650G	N30G	7.78	8.24	3/13/2020
SRR11494585	S	nonsynonymous	A21651G	N30G	7.78	8.28	3/13/2020

SRR11494585	S	nonsynonymous	T21652G	N30G	7.78	8.30	3/13/2020
SRR11494585	S	nonsynonymous	T21664A	R34P	8.58	9.78	3/13/2020
SRR11494585	S	nonsynonymous	G21663C	R34P	8.58	8.92	3/13/2020
SRR11494585	S	nonsynonymous	C21658A	F32Stop	8.36	9.86	3/13/2020
SRR11494585	S	nonsynonymous	T21657A	F32Stop	8.36	8.26	3/13/2020
SRR11494585	S	nonsynonymous	C21654A	S31Stop	7.38	8.24	3/13/2020
SRR11494585	S	nonsynonymous	T21655A	S31Stop	7.38	8.37	3/13/2020
SRR11494567	S	nonsynonymous	C21642T	A27V	18.97	18.49	3/15/2020
SRR11494567	S	nonsynonymous	G21665C	G35R	6.46	5.92	3/15/2020
SRR11494671	S	nonsynonymous	G24933T	G1124V	7.47	6.82	3/22/2020
SRR11494590	S	nonsynonymous	A22623G	N354S	21.12	21.92	3/13/2020
SRR11494590	S	nonsynonymous	G21665C	G35R	7.11	6.65	3/13/2020
SRR11494590	S	nonsynonymous	A21650G	N30G	5.07	5.37	3/13/2020
SRR11494590	S	nonsynonymous	T21652G	N30G	5.07	5.36	3/13/2020
SRR11494590	S	nonsynonymous	A21651G	N30G	5.07	5.36	3/13/2020
SRR11494590	S	nonsynonymous	C21658A	F32Stop	5.93	6.97	3/13/2020
SRR11494590	S	nonsynonymous	T21657A	F32Stop	5.93	5.72	3/13/2020
SRR11494590	S	nonsynonymous	G21663C	R34P	6.38	6.19	3/13/2020
SRR11494590	S	nonsynonymous	T21664A	R34P	6.38	7.92	3/13/2020
SRR11494590	S	nonsynonymous	C21654A	S31Stop	5.11	5.95	3/13/2020
SRR11494590	S	nonsynonymous	T21655A	S31Stop	5.11	5.46	3/13/2020
SRR11494555	S	nonsynonymous	T21655A	S31Stop	5.20	6.03	3/16/2020
SRR11494555	S	nonsynonymous	C21654A	S31Stop	5.20	5.72	3/16/2020
SRR11494555	S	nonsynonymous	T21657A	F32Stop	5.52	6.55	3/16/2020
SRR11494555	S	nonsynonymous	C21658A	F32Stop	5.52	7.08	3/16/2020
SRR11494555	S	nonsynonymous	G21665C	G35R	6.58	6.68	3/16/2020
SRR11494569	S	nonsynonymous	G21665C	G35R	5.60	5.06	3/14/2020
SRR11494530	S	nonsynonymous	G21665C	G35R	6.32	6.68	3/16/2020
SRR11494708	S	nonsynonymous	G21663C	R34P	5.15	4.77	3/18/2020
SRR11494708	S	nonsynonymous	T21664A	R34P	5.15	4.79	3/18/2020
SRR11494708	S	nonsynonymous	T21657A	F32Stop	5.44	5.23	3/18/2020
SRR11494708	S	nonsynonymous	C21658A	F32Stop	5.44	5.42	3/18/2020
SRR11494708	S	nonsynonymous	T21655A	S31Stop	5.36	5.26	3/18/2020
SRR11494708	S	nonsynonymous	C21654A	S31Stop	5.36	4.82	3/18/2020
SRR11494708	S	nonsynonymous	G21665C	G35R	5.31	5.01	3/18/2020
SRR11494615	S	nonsynonymous	G21665C	G35R	5.80	6.07	03/11/2020
SRR11494618	S	nonsynonymous	A21650G	N30G	5.14	5.29	03/10/2020
SRR11494618	S	nonsynonymous	T21652G	N30G	5.14	5.54	03/10/2020
SRR11494618	S	nonsynonymous	A21651G	N30G	5.14	5.33	03/10/2020
SRR11494618	S	nonsynonymous	G21665C	G35R	5.90	6.10	03/10/2020
SRR11494618	S	nonsynonymous	C21658A	F32Stop	6.02	6.18	03/10/2020
SRR11494618	S	nonsynonymous	T21657A	F32Stop	6.02	5.47	03/10/2020
SRR11494618	S	nonsynonymous	G21663C	R34P	5.18	5.74	03/10/2020
SRR11494618	S	nonsynonymous	T21664A	R34P	5.18	5.83	03/10/2020
SRR11494618	S	nonsynonymous	C21654A	S31Stop	5.00	5.36	03/10/2020
SRR11494618	S	nonsynonymous	T21655A	S31Stop	5.00	5.47	03/10/2020

SRR11494751	S	nonsynonymous	G21665C	G35R	6.49	6.04	03/09/2020
SRR11494751	S	nonsynonymous	C21658A	F32Stop	5.72	5.95	03/09/2020
SRR11494751	S	nonsynonymous	T21657A	F32Stop	5.72	5.29	03/09/2020
SRR11494645	S	nonsynonymous	G24557T	G999C	10.64	10.63	3/23/2020
SRR11494645	S	nonsynonymous	G22899T	G446V	13.45	12.26	3/23/2020
SRR11494645	S	nonsynonymous	G23343T	G594V	7.39	6.68	3/23/2020
SRR11494645	S	nonsynonymous	G24933T	G1124V	37.12	35.42	3/23/2020
SRR11494645	S	nonsynonymous	G22257T	G232V	6.79	6.45	3/23/2020
SRR11494570	S	nonsynonymous	T21664A	R34P	5.04	4.18	3/14/2020
SRR11494570	S	nonsynonymous	G21663C	R34P	5.04	3.83	3/14/2020
SRR11494570	S	nonsynonymous	G21665C	G35R	5.78	4.64	3/14/2020
SRR11494570	S	nonsynonymous	A23984G	D808D	38.06	38.43	3/14/2020
SRR11494548	S	nonsynonymous	A21651G	N30G	6.57	6.04	1/31/2020
SRR11494548	S	nonsynonymous	T21652G	N30G	6.57	6.05	1/31/2020
SRR11494548	S	nonsynonymous	A21650G	N30G	6.57	6.03	1/31/2020
SRR11494548	S	nonsynonymous	G21665C	G35R	7.44	6.89	1/31/2020
SRR11494548	S	nonsynonymous	C21658A	F32Stop	6.74	6.81	1/31/2020
SRR11494548	S	nonsynonymous	T21657A	F32Stop	6.74	6.59	1/31/2020
SRR11494548	S	nonsynonymous	T21664A	R34P	6.86	6.50	1/31/2020
SRR11494548	S	nonsynonymous	G21663C	R34P	6.86	6.45	1/31/2020
SRR11494548	S	nonsynonymous	C21654A	S31Stop	6.43	6.45	1/31/2020
SRR11494548	S	nonsynonymous	T21655A	S31Stop	6.43	6.62	1/31/2020
SRR11578357	S	synonymous	T22087C	F175	5.31	4.74	3/30/2020
SRR11494557	S	nonsynonymous	C24320A	Q920K	5.27	6.76	3/15/2020
SRR11494557	S	nonsynonymous	C24258T	A899V	8.37	7.37	3/15/2020
SRR11494587	S	nonsynonymous	G21665C	G35R	5.76	5.32	3/13/2020
SRR11494587	S	nonsynonymous	T21655A	S31Stop	5.02	5.05	3/13/2020
SRR11494587	S	nonsynonymous	C21654A	S31Stop	5.02	5.05	3/13/2020
SRR11494587	S	nonsynonymous	T21664A	R34P	5.14	5.75	3/13/2020
SRR11494587	S	nonsynonymous	G21663C	R34P	5.14	5.20	3/13/2020
SRR11494591	S	nonsynonymous	G21665C	G35R	6.13	5.40	3/13/2020
SRR11494455	S	nonsynonymous	T21657A	F32Stop	5.02	5.59	03/09/2020
SRR11494455	S	nonsynonymous	C21658A	F32Stop	5.02	5.79	03/09/2020
SRR11494455	S	nonsynonymous	G21665C	G35R	6.05	5.25	03/09/2020
SRR11494486	S	nonsynonymous	G21663C	R34P	7.96	6.97	3/17/2020
SRR11494486	S	nonsynonymous	T21664A	R34P	7.96	7.55	3/17/2020
SRR11494486	S	nonsynonymous	G21665C	G35R	8.64	7.53	3/17/2020
SRR11494486	S	nonsynonymous	C21707T	H49Y	7.08	6.58	3/17/2020
SRR11494486	S	nonsynonymous	G22464A	C301Y	34.30	5.59	3/17/2020
SRR11494486	S	nonsynonymous	T22465C	C301Y	34.30	4.40	3/17/2020
SRR11494486	S	nonsynonymous	T21657A	F32Stop	6.93	6.89	3/17/2020
SRR11494486	S	nonsynonymous	C21658A	F32Stop	6.93	7.34	3/17/2020
SRR11494486	S	synonymous	T21994C	Y144	5.71	5.15	3/17/2020
SRR11494486	S	nonsynonymous	G21606T	C15F	5.30	5.44	3/17/2020
SRR11494565	S	nonsynonymous	G21665C	G35R	5.64	5.17	3/15/2020
SRR11494565	S	nonsynonymous	G25049T	D1163Y	11.44	15.45	3/15/2020

SRR11494459	S	nonsynonymous	G21665C	G35R	6.19	6.22	3/19/2020
SRR11494607	S	nonsynonymous	C21654A	S31Stop	5.41	4.66	03/11/2020
SRR11494607	S	nonsynonymous	T21655A	S31Stop	5.41	4.62	03/11/2020
SRR11494607	S	nonsynonymous	G21665C	G35R	6.77	4.96	03/11/2020
SRR11494607	S	nonsynonymous	T21652G	N30G	5.32	4.49	03/11/2020
SRR11494607	S	nonsynonymous	A21651G	N30G	5.32	4.49	03/11/2020
SRR11494607	S	nonsynonymous	A21650G	N30G	5.32	4.63	03/11/2020
SRR11494478	S	synonymous	A22243G	V227	8.47	8.22	03/08/2020
SRR11494578	S	nonsynonymous	T21657A	F32Stop	8.79	9.06	3/14/2020
SRR11494578	S	nonsynonymous	C21658A	F32Stop	8.79	9.88	3/14/2020
SRR11494578	S	nonsynonymous	G21663C	R34P	8.45	9.24	3/14/2020
SRR11494578	S	nonsynonymous	T21664A	R34P	8.45	10.14	3/14/2020
SRR11494578	S	nonsynonymous	C21654A	S31Stop	7.73	9.09	3/14/2020
SRR11494578	S	nonsynonymous	T21655A	S31Stop	7.73	9.11	3/14/2020
SRR11494578	S	nonsynonymous	T21652G	N30G	8.18	8.88	3/14/2020
SRR11494578	S	nonsynonymous	A21651G	N30G	8.18	8.67	3/14/2020
SRR11494578	S	nonsynonymous	A21650G	N30G	8.18	8.48	3/14/2020
SRR11494578	S	nonsynonymous	G21665C	G35R	9.91	9.62	3/14/2020
SRR11494562	S	nonsynonymous	G21665C	G35R	5.54	4.87	3/15/2020
SRR11494617	S	nonsynonymous	T21655A	S31Stop	5.04	5.03	03/10/2020
SRR11494617	S	nonsynonymous	C21654A	S31Stop	5.04	5.06	03/10/2020
SRR11494617	S	nonsynonymous	G21663C	R34P	5.51	5.37	03/10/2020
SRR11494617	S	nonsynonymous	T21664A	R34P	5.51	5.61	03/10/2020
SRR11494617	S	nonsynonymous	A21651G	N30G	5.19	4.74	03/10/2020
SRR11494617	S	nonsynonymous	T21652G	N30G	5.19	4.74	03/10/2020
SRR11494617	S	nonsynonymous	A21650G	N30G	5.19	4.70	03/10/2020
SRR11494617	S	nonsynonymous	G21665C	G35R	6.58	5.46	03/10/2020
SRR11494524	S	nonsynonymous	G24914C	D1118H	42.77	42.06	3/17/2020
SRR11494559	S	nonsynonymous	T21657A	F32Stop	6.17	6.26	3/15/2020
SRR11494559	S	nonsynonymous	C21658A	F32Stop	6.17	6.70	3/15/2020
SRR11494559	S	nonsynonymous	G21663C	R34P	7.20	6.71	3/15/2020
SRR11494559	S	nonsynonymous	T21664A	R34P	7.20	6.74	3/15/2020
SRR11494559	S	nonsynonymous	G23403A	D614D	9.78	9.07	3/15/2020
SRR11494559	S	nonsynonymous	C21654A	S31Stop	5.54	5.77	3/15/2020
SRR11494559	S	nonsynonymous	T21655A	S31Stop	5.54	6.06	3/15/2020
SRR11494559	S	nonsynonymous	G21665C	G35R	7.53	6.78	3/15/2020
SRR11494559	S	nonsynonymous	A21651G	N30G	6.14	5.76	3/15/2020
SRR11494559	S	nonsynonymous	T21652G	N30G	6.14	5.76	3/15/2020
SRR11494559	S	nonsynonymous	A21650G	N30G	6.14	5.74	3/15/2020
SRR11494605	S	nonsynonymous	G21665C	G35R	5.33	4.05	03/12/2020
SRR11494465	S	nonsynonymous	G21665C	G35R	6.89	5.94	3/17/2020
SRR11494465	S	nonsynonymous	A21651G	N30G	5.54	4.80	3/17/2020
SRR11494465	S	nonsynonymous	T21652G	N30G	5.54	4.81	3/17/2020
SRR11494465	S	nonsynonymous	A21650G	N30G	5.54	5.22	3/17/2020
SRR11494465	S	nonsynonymous	C21654A	S31Stop	5.59	5.22	3/17/2020
SRR11494465	S	nonsynonymous	T21655A	S31Stop	5.59	5.10	3/17/2020

SRR11494465	S	nonsynonymous	G21663C	R34P	5.36	5.23	3/17/2020
SRR11494465	S	nonsynonymous	T21664A	R34P	5.36	6.04	3/17/2020
SRR11494465	S	nonsynonymous	T21657A	F32Stop	5.98	5.31	3/17/2020
SRR11494465	S	nonsynonymous	C21658A	F32Stop	5.98	5.97	3/17/2020
SRR11494657	S	nonsynonymous	G23288T	V576F	6.07	6.84	3/23/2020
SRR11494657	S	nonsynonymous	G24557T	G999C	15.52	15.27	3/23/2020
SRR11494657	S	nonsynonymous	G23343T	G594V	10.19	9.14	3/23/2020
SRR11494657	S	nonsynonymous	G22899T	G446V	12.66	11.80	3/23/2020
SRR11494657	S	nonsynonymous	G25352T	V1264L	5.70	5.28	3/23/2020
SRR11578317	S	nonsynonymous	T23126G	A522A	6.36	5.56	3/27/2020
SRR11494599	S	synonymous	A22606G	A348	6.77	7.25	03/12/2020
SRR11494621	S	nonsynonymous	T21657A	F32Stop	5.15	5.47	03/10/2020
SRR11494621	S	nonsynonymous	C21658A	F32Stop	5.15	5.67	03/10/2020
SRR11494621	S	nonsynonymous	G21665C	G35R	5.56	6.05	03/10/2020
SRR11621998	S	nonsynonymous	T23311G	E583E	5.21	5.20	04/03/2020
SRR11494656	S	nonsynonymous	G24933T	G1124V	11.79	12.34	3/23/2020
SRR11494582	S	nonsynonymous	G21665C	G35R	5.69	4.76	3/13/2020
SRR11494609	S	nonsynonymous	G21665C	G35R	8.31	5.36	03/11/2020
SRR11494609	S	nonsynonymous	A21650G	N30G	6.17	4.75	03/11/2020
SRR11494609	S	nonsynonymous	A21651G	N30G	6.17	4.77	03/11/2020
SRR11494609	S	nonsynonymous	T21652G	N30G	6.17	4.97	03/11/2020
SRR11494609	S	nonsynonymous	C21654A	S31Stop	6.68	5.18	03/11/2020
SRR11494609	S	nonsynonymous	T21655A	S31Stop	6.68	5.55	03/11/2020
SRR11494609	S	nonsynonymous	T21657A	F32Stop	6.75	5.55	03/11/2020
SRR11494609	S	nonsynonymous	C21658A	F32Stop	6.75	5.55	03/11/2020
SRR11494609	S	nonsynonymous	G21663C	R34P	6.82	5.26	03/11/2020
SRR11494609	S	nonsynonymous	T21664A	R34P	6.82	5.51	03/11/2020
SRR11494581	S	nonsynonymous	T21664A	R34P	5.99	5.39	3/13/2020
SRR11494581	S	nonsynonymous	G21663C	R34P	5.99	5.32	3/13/2020
SRR11494581	S	nonsynonymous	C21658A	F32Stop	5.43	6.38	3/13/2020
SRR11494581	S	nonsynonymous	T21657A	F32Stop	5.43	5.09	3/13/2020
SRR11494581	S	nonsynonymous	G21665C	G35R	6.25	5.43	3/13/2020
SRR11494603	S	nonsynonymous	G21665C	G35R	6.29	6.70	03/12/2020
SRR11494603	S	nonsynonymous	G21663C	R34P	6.02	6.49	03/12/2020
SRR11494603	S	nonsynonymous	T21664A	R34P	6.02	6.54	03/12/2020
SRR11494603	S	nonsynonymous	C21658A	F32Stop	5.52	8.16	03/12/2020
SRR11494603	S	nonsynonymous	T21657A	F32Stop	5.52	6.32	03/12/2020
SRR11494603	S	nonsynonymous	C21654A	S31Stop	5.11	6.24	03/12/2020
SRR11494603	S	nonsynonymous	T21655A	S31Stop	5.11	6.41	03/12/2020
SRR11494592	S	nonsynonymous	T21655A	S31Stop	5.04	4.83	3/13/2020
SRR11494592	S	nonsynonymous	C21654A	S31Stop	5.04	4.52	3/13/2020
SRR11494592	S	nonsynonymous	T21657A	F32Stop	5.64	4.81	3/13/2020
SRR11494592	S	nonsynonymous	C21658A	F32Stop	5.64	5.18	3/13/2020
SRR11494592	S	nonsynonymous	G21663C	R34P	6.18	5.03	3/13/2020
SRR11494592	S	nonsynonymous	T21664A	R34P	6.18	5.12	3/13/2020
SRR11494592	S	nonsynonymous	G21665C	G35R	6.67	5.14	3/13/2020

SRR11494592	S	nonsynonymous	A21650G	N30G	5.18	4.36	3/13/2020
SRR11494592	S	nonsynonymous	A21651G	N30G	5.18	4.52	3/13/2020
SRR11494592	S	nonsynonymous	T21652G	N30G	5.18	4.52	3/13/2020
SRR11494662	S	nonsynonymous	G22442T	D294Y	5.33	4.83	3/23/2020
SRR11494537	S	nonsynonymous	G21663C	R34P	6.87	9.20	02/02/2020
SRR11494537	S	nonsynonymous	T21664A	R34P	6.87	10.57	02/02/2020
SRR11494537	S	nonsynonymous	C21658A	F32Stop	6.87	9.44	02/02/2020
SRR11494537	S	nonsynonymous	T21657A	F32Stop	6.87	8.24	02/02/2020
SRR11494537	S	nonsynonymous	T21655A	S31Stop	6.64	8.33	02/02/2020
SRR11494537	S	nonsynonymous	C21654A	S31Stop	6.64	8.22	02/02/2020
SRR11494537	S	nonsynonymous	T21652G	N30G	6.86	7.95	02/02/2020
SRR11494537	S	nonsynonymous	A21651G	N30G	6.86	7.95	02/02/2020
SRR11494537	S	nonsynonymous	A21650G	N30G	6.86	7.86	02/02/2020
SRR11494537	S	nonsynonymous	G21665C	G35R	8.30	9.48	02/02/2020
SRR11494566	S	nonsynonymous	A21650G	N30G	6.67	7.50	3/15/2020
SRR11494566	S	nonsynonymous	A21651G	N30G	6.67	7.50	3/15/2020
SRR11494566	S	nonsynonymous	T21652G	N30G	6.67	7.50	3/15/2020
SRR11494566	S	nonsynonymous	G21665C	G35R	8.40	9.22	3/15/2020
SRR11494566	S	nonsynonymous	C21658A	F32Stop	7.36	8.57	3/15/2020
SRR11494566	S	nonsynonymous	T21657A	F32Stop	7.36	8.03	3/15/2020
SRR11494566	S	nonsynonymous	T21664A	R34P	7.89	9.14	3/15/2020
SRR11494566	S	nonsynonymous	G21663C	R34P	7.89	8.13	3/15/2020
SRR11494566	S	nonsynonymous	C21654A	S31Stop	6.12	7.73	3/15/2020
SRR11494566	S	nonsynonymous	T21655A	S31Stop	6.12	8.07	3/15/2020
SRR11494638	S	synonymous	T23557C	P665	9.84	9.74	3/23/2020
SRR11494638	S	nonsynonymous	G24857A	G1099S	13.69	13.28	3/23/2020
SRR11494638	S	synonymous	T22918G	L452	31.68	33.62	3/23/2020
SRR11494561	S	nonsynonymous	T24847A	F1095L	33.34	33.16	3/15/2020
SRR11494727	S	nonsynonymous	T21657A	F32Stop	6.04	7.04	03/09/2020
SRR11494727	S	nonsynonymous	C21658A	F32Stop	6.04	7.78	03/09/2020
SRR11494727	S	nonsynonymous	A21650G	N30G	5.93	6.47	03/09/2020
SRR11494727	S	nonsynonymous	A21651G	N30G	5.93	6.67	03/09/2020
SRR11494727	S	nonsynonymous	T21652G	N30G	5.93	6.67	03/09/2020
SRR11494727	S	nonsynonymous	T21664A	R34P	6.14	7.99	03/09/2020
SRR11494727	S	nonsynonymous	G21663C	R34P	6.14	7.26	03/09/2020
SRR11494727	S	nonsynonymous	G21665C	G35R	6.73	7.73	03/09/2020
SRR11494600	S	nonsynonymous	T21655A	S31Stop	5.50	6.92	03/12/2020
SRR11494600	S	nonsynonymous	C21654A	S31Stop	5.50	6.79	03/12/2020
SRR11494600	S	nonsynonymous	T21657A	F32Stop	5.60	6.83	03/12/2020
SRR11494600	S	nonsynonymous	C21658A	F32Stop	5.60	7.79	03/12/2020
SRR11494600	S	nonsynonymous	T21664A	R34P	5.42	6.70	03/12/2020
SRR11494600	S	nonsynonymous	G21663C	R34P	5.42	6.76	03/12/2020
SRR11494600	S	nonsynonymous	G21665C	G35R	6.62	7.14	03/12/2020
SRR11494529	S	synonymous	T25207C	Y1215	13.21	12.30	3/17/2020
SRR11494551	S	synonymous	C22264T	N234	12.58	11.34	3/16/2020
SRR11494553	S	nonsynonymous	G21665C	G35R	5.10	4.61	3/16/2020

SRR11494588	S	nonsynonymous	G21665C	G35R	6.94	5.99	3/13/2020
SRR11494588	S	nonsynonymous	C21654A	S31Stop	5.70	5.46	3/13/2020
SRR11494588	S	nonsynonymous	T21655A	S31Stop	5.70	5.65	3/13/2020
SRR11494588	S	nonsynonymous	T21657A	F32Stop	6.04	5.75	3/13/2020
SRR11494588	S	nonsynonymous	C21658A	F32Stop	6.04	6.79	3/13/2020
SRR11494588	S	nonsynonymous	T21664A	R34P	6.47	5.84	3/13/2020
SRR11494588	S	nonsynonymous	G21663C	R34P	6.47	5.56	3/13/2020
SRR11494593	S	nonsynonymous	A23631G	Q690R	5.78	6.12	03/12/2020
SRR11494589	S	nonsynonymous	G21665C	G35R	5.45	3.87	3/13/2020
SRR11494589	S	nonsynonymous	T21657A	F32Stop	5.05	3.47	3/13/2020
SRR11494589	S	nonsynonymous	C21658A	F32Stop	5.05	3.37	3/13/2020
SRR11494589	S	nonsynonymous	T21664A	R34P	5.10	3.87	3/13/2020
SRR11494589	S	nonsynonymous	G21663C	R34P	5.10	3.52	3/13/2020
SRR11494610	S	nonsynonymous	T21655A	S31Stop	7.30	8.35	03/11/2020
SRR11494610	S	nonsynonymous	C21654A	S31Stop	7.30	8.55	03/11/2020
SRR11494610	S	nonsynonymous	G21663C	R34P	7.53	7.94	03/11/2020
SRR11494610	S	nonsynonymous	T21664A	R34P	7.53	8.50	03/11/2020
SRR11494610	S	nonsynonymous	C21658A	F32Stop	7.84	9.53	03/11/2020
SRR11494610	S	nonsynonymous	T21657A	F32Stop	7.84	8.49	03/11/2020
SRR11494610	S	nonsynonymous	A21650G	N30G	5.87	7.55	03/11/2020
SRR11494610	S	nonsynonymous	T21652G	N30G	5.87	7.74	03/11/2020
SRR11494610	S	nonsynonymous	A21651G	N30G	5.87	7.35	03/11/2020
SRR11494610	S	nonsynonymous	G21665C	G35R	8.23	8.71	03/11/2020
SRR11494576	S	nonsynonymous	G21665C	G35R	5.65	5.88	3/14/2020
SRR11494576	S	nonsynonymous	G21663C	R34P	5.57	5.46	3/14/2020
SRR11494576	S	nonsynonymous	T21664A	R34P	5.57	5.83	3/14/2020
SRR11494604	S	synonymous	G22468T	T302	5.06	8.76	03/12/2020
SRR11494490	S	nonsynonymous	G21665C	G35R	6.18	7.27	03/07/2020
SRR11494490	S	nonsynonymous	A21650G	N30G	5.15	5.54	03/07/2020
SRR11494490	S	nonsynonymous	T21652G	N30G	5.15	5.98	03/07/2020
SRR11494490	S	nonsynonymous	A21651G	N30G	5.15	5.77	03/07/2020
SRR11494490	S	nonsynonymous	G21663C	R34P	5.17	6.17	03/07/2020
SRR11494490	S	nonsynonymous	T21664A	R34P	5.17	7.51	03/07/2020
SRR11494490	S	nonsynonymous	C21658A	F32Stop	5.71	7.62	03/07/2020
SRR11494490	S	nonsynonymous	T21657A	F32Stop	5.71	5.84	03/07/2020
SRR11494490	S	nonsynonymous	T21655A	S31Stop	5.08	5.87	03/07/2020
SRR11494490	S	nonsynonymous	C21654A	S31Stop	5.08	5.78	03/07/2020
SRR11494568	S	synonymous	A23335T	S591	38.99	38.47	3/14/2020
SRR11494568	S	nonsynonymous	A21650G	N30G	5.94	6.20	3/14/2020
SRR11494568	S	nonsynonymous	A21651G	N30G	5.94	6.10	3/14/2020
SRR11494568	S	nonsynonymous	T21652G	N30G	5.94	6.26	3/14/2020
SRR11494568	S	nonsynonymous	G21665C	G35R	7.34	6.82	3/14/2020
SRR11494568	S	nonsynonymous	T21664A	R34P	6.22	7.00	3/14/2020
SRR11494568	S	nonsynonymous	G21663C	R34P	6.22	6.60	3/14/2020
SRR11494568	S	nonsynonymous	C21658A	F32Stop	6.36	6.96	3/14/2020
SRR11494568	S	nonsynonymous	T21657A	F32Stop	6.36	6.44	3/14/2020

SRR11494568	S	nonsynonymous	T21655A	S31Stop	6.08	6.46	3/14/2020
SRR11494568	S	nonsynonymous	C21654A	S31Stop	6.08	6.31	3/14/2020
SRR11494568	S	nonsynonymous	T25236G	I1225S	12.83	12.95	3/14/2020
SRR11494560	S	nonsynonymous	G21665C	G35R	5.01	4.17	3/15/2020
SRR11494612	S	nonsynonymous	G21665C	G35R	5.52	5.63	03/11/2020
SRR11494683	S	nonsynonymous	A21651G	N30G	5.84	4.67	3/22/2020
SRR11494683	S	nonsynonymous	T21652G	N30G	5.84	4.85	3/22/2020
SRR11494683	S	nonsynonymous	A21650G	N30G	5.84	4.49	3/22/2020
SRR11494683	S	nonsynonymous	G21665C	G35R	6.87	5.27	3/22/2020
SRR11494683	S	nonsynonymous	G21663C	R34P	6.67	5.12	3/22/2020
SRR11494683	S	nonsynonymous	T21664A	R34P	6.67	5.37	3/22/2020
SRR11494683	S	nonsynonymous	C21658A	F32Stop	6.36	6.92	3/22/2020
SRR11494683	S	nonsynonymous	T21657A	F32Stop	6.36	5.26	3/22/2020
SRR11494683	S	nonsynonymous	T21655A	S31Stop	6.17	4.82	3/22/2020
SRR11494683	S	nonsynonymous	C21654A	S31Stop	6.17	4.99	3/22/2020
SRR11494559	nucleocapsid	nonsynonymous	T28854C	S194S	8.25	8.14	3/15/2020
SRR11578318	nucleocapsid	nonsynonymous	G29017T	K248N	8.21	8.70	3/27/2020
SRR11578318	nucleocapsid	nonsynonymous	C29003A	Q244K	7.92	8.99	3/27/2020
SRR11494618	nucleocapsid	nonsynonymous	A29433G	K387R	9.73	10.29	03/10/2020
SRR11494529	nucleocapsid	nonsynonymous	C29171T	H300Y	6.87	6.78	3/17/2020
SRR11494494	nucleocapsid	nonsynonymous	C28868T	P199S	6.64	6.92	3/18/2020
SRR11494591	nucleocapsid	nonsynonymous	A28868C	P199P	7.63	8.22	3/13/2020
SRR11494585	nucleocapsid	nonsynonymous	G28808T	G179C	12.37	11.40	3/13/2020
SRR11494585	nucleocapsid	nonsynonymous	G28719T	R149L	6.28	5.72	3/13/2020
SRR11494537	nucleocapsid	nonsynonymous	G29405A	E378K	5.73	5.54	02/02/2020
SRR11494537	nucleocapsid	nonsynonymous	T29068A	T265P	7.14	6.62	02/02/2020
SRR11494537	nucleocapsid	nonsynonymous	A29066C	T265P	7.14	6.69	02/02/2020
SRR11494627	nucleocapsid	synonymous	G28939T	L222	5.17	5.21	3/24/2020
SRR11494594	nucleocapsid	synonymous	G28939A	L222	6.59	5.70	03/12/2020
SRR11494665	nucleocapsid	nonsynonymous	G29049A	R259Q	22.22	15.38	3/22/2020
SRR11494665	nucleocapsid	nonsynonymous	A29039T	K256Stop	19.05	16.33	3/22/2020
SRR11494665	nucleocapsid	nonsynonymous	C29057A	R262I	17.31	7.02	3/22/2020
SRR11494665	nucleocapsid	nonsynonymous	G29058T	R262I	17.31	12.28	3/22/2020
SRR11494665	nucleocapsid	synonymous	A29053G	Q260	20.41	9.09	3/22/2020
SRR11494665	nucleocapsid	nonsynonymous	A29060T	T263S	10.00	6.78	3/22/2020
SRR11494665	nucleocapsid	nonsynonymous	A29054C	K261Q	19.61	7.27	3/22/2020
SRR11494665	nucleocapsid	nonsynonymous	A29056G	K261Q	19.61	6.90	3/22/2020
SRR11494548	nucleocapsid	nonsynonymous	G29017C	K248N	12.17	3.90	1/31/2020
SRR11494548	nucleocapsid	synonymous	A28780G	K169	6.59	5.99	1/31/2020
SRR11494486	nucleocapsid	nonsynonymous	G28985A	G238S	11.72	11.54	3/17/2020
SRR11494606	nucleocapsid	synonymous	T28756C	L161	20.07	20.32	03/11/2020
SRR11578164	nucleocapsid	nonsynonymous	G28559T	G96C	8.40	7.35	3/25/2020
SRR11578164	nucleocapsid	nonsynonymous	G29321T	V350F	5.83	4.97	3/25/2020
SRR11494638	nucleocapsid	nonsynonymous	G28881A	R203K	6.73	6.70	3/23/2020
SRR11494638	nucleocapsid	nonsynonymous	G28882A	R203K	6.73	6.96	3/23/2020
SRR11494638	nucleocapsid	nonsynonymous	G28883C	G204R	8.27	7.50	3/23/2020

SRR11494657	nucleocapsid	nonsynonymous	G28559T	G96C	8.96	8.07	3/23/2020
SRR11494645	nucleocapsid	nonsynonymous	G28559T	G96C	8.29	7.94	3/23/2020
SRR11494645	nucleocapsid	nonsynonymous	G28517T	D82Y	8.12	7.45	3/23/2020
SRR11494612	nucleocapsid	synonymous	T28315C	R14	16.64	15.90	03/11/2020
SRR11494577	nucleocapsid	nonsynonymous	T28978C	S235T	10.00	3.69	3/14/2020
SRR11494577	nucleocapsid	nonsynonymous	T28976A	S235T	10.00	7.91	3/14/2020
SRR11494515	nucleocapsid	nonsynonymous	C28367T	R32C	7.15	7.17	3/18/2020
SRR11494616	nucleocapsid	nonsynonymous	A29117G	T282A	5.90	6.04	03/11/2020
SRR11494623	nucleocapsid	nonsynonymous	G28338A	D22D	27.68	26.31	3/24/2020
SRR11494623	nucleocapsid	synonymous	T29212G	A313	26.02	25.98	3/24/2020
